# An exceptional phytoplankton bloom in the southeast Madagascar Sea driven by African dust deposition

**DOI:** 10.1093/pnasnexus/pgae386

**Published:** 2024-10-01

**Authors:** John A Gittings, Giorgio Dall’Olmo, Weiyi Tang, Joan Llort, Fatma Jebri, Eleni Livanou, Francesco Nencioli, Sofia Darmaraki, Iason Theodorou, Robert J W Brewin, Meric Srokosz, Nicolas Cassar, Dionysios E Raitsos

**Affiliations:** Department of Biology, National and Kapodistrian University of Athens, Athens 15784, Greece; Sezione di Oceanografia, Istituto Nazionale di Oceanografia e Geofisica Sperimentale—OGS, Borgo Grotta Gigante, Trieste 34010, Italy; Department of Geosciences, Princeton University; Guyot Hall, Princeton, NJ 08544, USA; Barcelona Supercomputing Center, Barcelona 08034, Spain; National Oceanography Centre, Southampton SO14 3ZH, United Kingdom; Department of Biology, National and Kapodistrian University of Athens, Athens 15784, Greece; Collecte Localisation Satellites, Ramonville-Saint-Agne 31520, France; Department of Biology, National and Kapodistrian University of Athens, Athens 15784, Greece; Department of Biology, National and Kapodistrian University of Athens, Athens 15784, Greece; Department of Earth and Environmental Science, Faculty of Environment, Science and Economy, Centre for Geography and Environmental Science, University of Exeter, Cornwall TR10 9FE, United Kingdom; National Oceanography Centre, Southampton SO14 3ZH, United Kingdom; Division of Earth and Climate Sciences, Nicholas School of the Environment, Duke University, Durham, NC 27708, USA; Department of Biology, National and Kapodistrian University of Athens, Athens 15784, Greece

## Abstract

Rising surface temperatures are projected to cause more frequent and intense droughts in the world's drylands. This can lead to land degradation, mobilization of soil particles, and an increase in dust aerosol emissions from arid and semi-arid regions. Dust aerosols are a key source of bio-essential nutrients, can be transported in the atmosphere over large distances, and ultimately deposited onto the ocean's surface, alleviating nutrient limitation and increasing oceanic primary productivity. Currently, the linkages between desertification, dust emissions and ocean fertilization remain poorly understood. Here, we show that dust emitted from Southern Africa was transported and deposited into the nutrient-limited surface waters southeast of Madagascar, which stimulated the strongest phytoplankton bloom of the last two decades during a period of the year when blooms are not expected. The conditions required for triggering blooms of this magnitude are anomalous, but current trends in air temperatures, aridity, and dust emissions in Southern Africa suggest that such events could become more probable in the future. Together with the recent findings on ocean fertilization by drought-induced megafires in Australia, our results point toward a potential link between global warming, drought, aerosol emissions, and ocean blooms.

**Significance Statement**

Dust aerosols are a key source of bio-essential nutrients, can be transported in the atmosphere over large distances, and deposited onto the ocean's surface, alleviating nutrient limitation, and increasing oceanic primary productivity. Linkages between dryland desertification, dust emissions, and ocean fertilization remain understudied. We show that desert dust emissions from drought-stricken Southern Africa were transported and deposited in the southwest Indian Ocean, stimulating the strongest phytoplankton bloom of the last two decades. The conditions required for triggering blooms of this magnitude are exceptional, yet current trends in air temperatures, aridity, and dust emissions in Southern Africa suggest that such mechanisms could become more frequent. Our results point toward a potential link between global warming, drought, aerosol emissions, and ocean blooms.

## Introduction

Anthropogenic warming has intensified extreme events, including droughts and heatwaves (1–3). Drylands comprise ∼41% of the global land area, are vulnerable to extreme drought, and are currently at risk of expanding desertification (4, 5). Vegetation loss in dry regions promotes the wind-driven mobilization of soil particles, enhancing atmospheric dust emissions (6). Dust aerosols are typically enriched in bio-essential nutrients, such as iron (Fe), nitrogen, and phosphorus (7, 8) and, when deposited over the ocean, can trigger substantial, but episodic increases in primary productivity (9–12).

In the Southern Hemisphere (SH), the collective drylands of Southern Africa constitute one of the major suppliers of dust to the iron-limited Southern Ocean and its peripheral regions (13, 14). Key dust-source areas include the Etosha and Makgadikgadi Pans in Namibia and Botswana, respectively (15–17), pans and ephemeral rivers in the coastal Namibian desert, as well as the South–Western Kalahari Pan belt (16). Dunefields in the Southern Kalahari Desert are predicted to mobilize following vegetation loss and could also become a potential source of dust capable of reaching the Southern Ocean (13, 18).

Southern Africa has been characterized as a hotspot of global climate change and current projections emphasize rising temperatures and increasing aridity (1, 19, 20). Prolonged and extreme multiyear droughts have occurred in Southern Africa over the last decade (21), culminating in the austral spring of 2019, which was amongst the driest in the last 40 years for parts of Zimbabwe, Namibia, Botswana, and South Africa (22). Approximately 90,000 livestock were lost in Namibia (23) and over 11 million people encountered remarkable levels of food insecurity (24). Temperature-driven extreme events during late 2019 were not limited to Southern Africa. Across the Indian Ocean, concurrent record-breaking megafires occurred in Australia, causing catastrophic environmental and economic impacts (25). An outcome of the Australian megafires was the subsequent wind-driven transport (26) and deposition of iron-rich aerosols, which triggered exceptionally widespread phytoplankton blooms thousands of kilometers away in the Southern Pacific Ocean (27).

We demonstrate that dust emissions from drought-stricken Southern African drylands stimulated an analogously massive bloom of marine phytoplankton off the Madagascar southeast coast in the Indian Ocean in late 2019. Taken together with the recent findings on the Australian megafires (27), our results suggest that the expected increase in aerosols associated with enhanced desertification could become an important source of nutrients for phytoplankton, potentially boosting atmospheric CO_2_ ocean uptake if they are deposited to the ocean's surface.

## Results and discussion

**Fig. 1.**
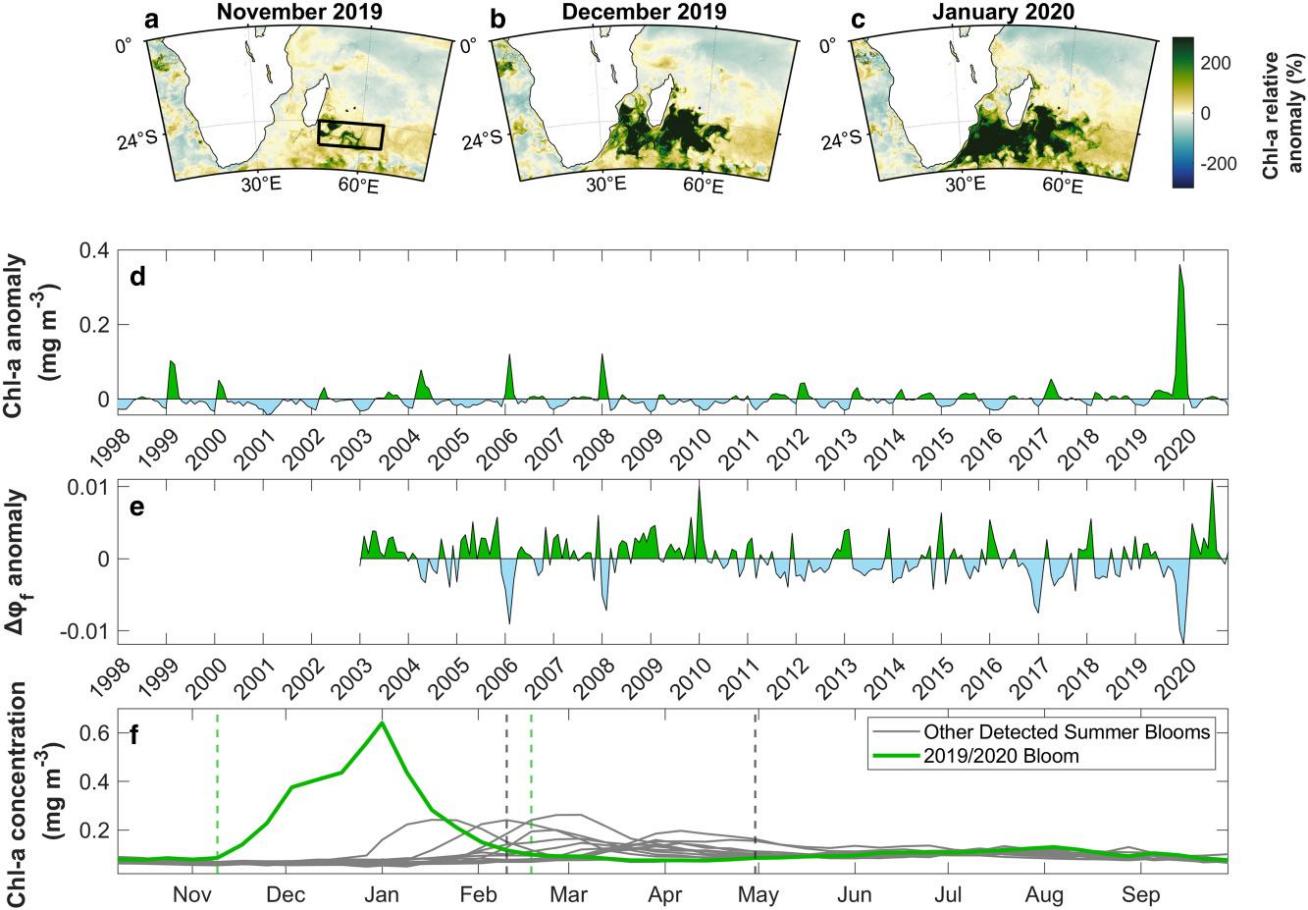
Magnitude and timing of the 2019–2020 South–East Madagascar phytoplankton bloom. (a to c) Monthly relative anomalies between 2019 November and 2020 January demonstrate the spatial development of Chl-a concentration, a proxy for phytoplankton biomass, during austral spring/summer. Relative anomalies are expressed as the % above the monthly climatological mean, relative to the period January 1998—December 2020. The black rectangle highlights the bloom area (24–30°S; 48–66°E) used for computing spatial averages (28, 29). (d) Monthly anomalies of Chl-a concentration (OC-CCI v6.0) averaged over the bloom area (see black rectangle in left panel of (a)), shown for the period between 1998 January and 2020 December. (e) Monthly anomalies of the chlorophyll fluorescence quantum yield (Δφ_f_, a proxy of iron-related stress) averaged over the bloom area (f) 8-day time series of Chl-a concentration alongside the timings of bloom initiation and termination (green vertical dashed lines) during the austral spring/summer of 2019/2020. The Chl-a time series for the remaining summer bloom years are shown in gray, alongside their corresponding climatological phenology metrics (bloom initiation and termination shown by the gray vertical dashed lines).

The 2019/2020 South–East Madagascar Bloom was remarkable with regards to both its timing and magnitude (Fig. 1). In 2019 November, the bloom developed as two mesoscale eddies located just southeast of Madagascar (30), characterized by Chlorophyll-a (Chl-a) concentrations that were at least 200% higher than the monthly climatological values (Fig. 1a). Strong eddy kinetic energy (EKE) in 2019 December enabled the diffusion of fertilized waters into the Mozambique Channel and Madagascar basin (Figs. 1b, c and S1). The monthly Chl-a anomaly spatially averaged over the bloom area (black rectangle in Fig. 1a) more than tripled in 2019 December (∼0.34 mg m^−3^), relative to summer blooms in other years (∼0.1 mg m^−3^, Fig. 1d), reaching concentrations that have never been observed over the entire 24-year satellite ocean color record. Satellite-derived monthly anomalies of primary production were substantially higher than climatological values between 2019 November and 2020 February, whilst the anomaly in satellite-based export production reached an unprecedented maximum in 2019 December (Fig. S2), supporting prior observations that the bloom area functioned as an oceanic carbon sink during this event (30). Not only was this bloom exceptional for its magnitude, but also because of when it occurred and how long it lasted. Phenological analyses (timing of phytoplankton growth) revealed that the bloom initiated 2.5 months earlier and lasted 3 weeks longer than previous Madagascar blooms in the austral summer (Fig. 1f).

Numerous hypotheses have been formulated to explain the onset of previous South–East Madagascar Blooms (28, 29, 31–35). Regions of the southwest Indian Ocean, adjacent to Madagascar, are suggested to be depleted in nitrate (30, 36) and iron (37, 38). Collectively, there is a consensus that these blooms initiate when stratification and temperatures increase, which are the optimal conditions for the proliferation of nitrogen-fixing diazotrophs (28–31, 39). Microscopy analyses conducted during earlier campaigns have revealed high abundances of *Trichodesmium* and/or diatom-diazotroph associations (e.g. *Richelia/Rhizosolenia*) in waters south and southeast of Madagascar (31, 39). In January 2020, in situ measurements of nitrogen fixation (N_2_) by micro-phytoplankton (>20 μm) confirmed that N_2_ fixation increased by a factor of 5 within the Madagascar bloom area, relative to measurements in the surrounding waters, supporting the presence of diazotrophs (30). A key limiting factor for the growth of diazotrophic phytoplankton is the availability of iron (Fe), an essential component of the nitrogenase enzyme that catalyzes nitrogen fixation (40). Iron stress in phytoplankton is known to induce an increase in chlorophyll fluorescence yields (Δφ_f_)—a relationship which has been demonstrated at regional and global scales (41–44). Clearly, the Δφ_f_ monthly anomaly reached an unprecedented minimum over the region in late-2019, indicating an abrupt relief in iron stress during the onset and development of the bloom (Fig. 1d, e).

Previous studies have suggested that the South–East Madagascar Bloom could be fertilized by iron-rich sediments advected from the south and east coasts of Madagascar (28, 33). We conducted an in-depth analysis of Lagrangian trajectories to quantify the potential contribution of advected nutrient-rich waters from the east coast of Madagascar and southeast Africa continental shelf (Figs. S3–S8, and Supplementary material). Within 60 days prior to the bloom initiation, ∼75% of water parcels we tracked to the bloom area did not originate from adjacent land masses. In other words, the contribution of coastal/shelf waters to particles found within the bloom region was minimal and comparable with previous nonbloom years (Supplementary material).

Alternative physical processes, such as vertical mixing and upwelling, can also supply iron and nutrients to the oceanic mixed layer. However, an in-depth analysis of the biophysical dynamics in the upper layer of the water column during the bloom demonstrated that the oceanographic physical settings were not anomalous relative to other years when blooms did not occur (Figs. S9 and S10, and Supplementary material). In addition, photosynthetically active radiation (PAR) within the mixed layer remained constant (∼20–40 E m^−2^ day^−1^) before and after the bloom, implying that it was not a limiting factor for growth (Fig. S11). Therefore, the anomalous magnitude and timing of this bloom suggest a different driving mechanism.

**Fig. 2.**
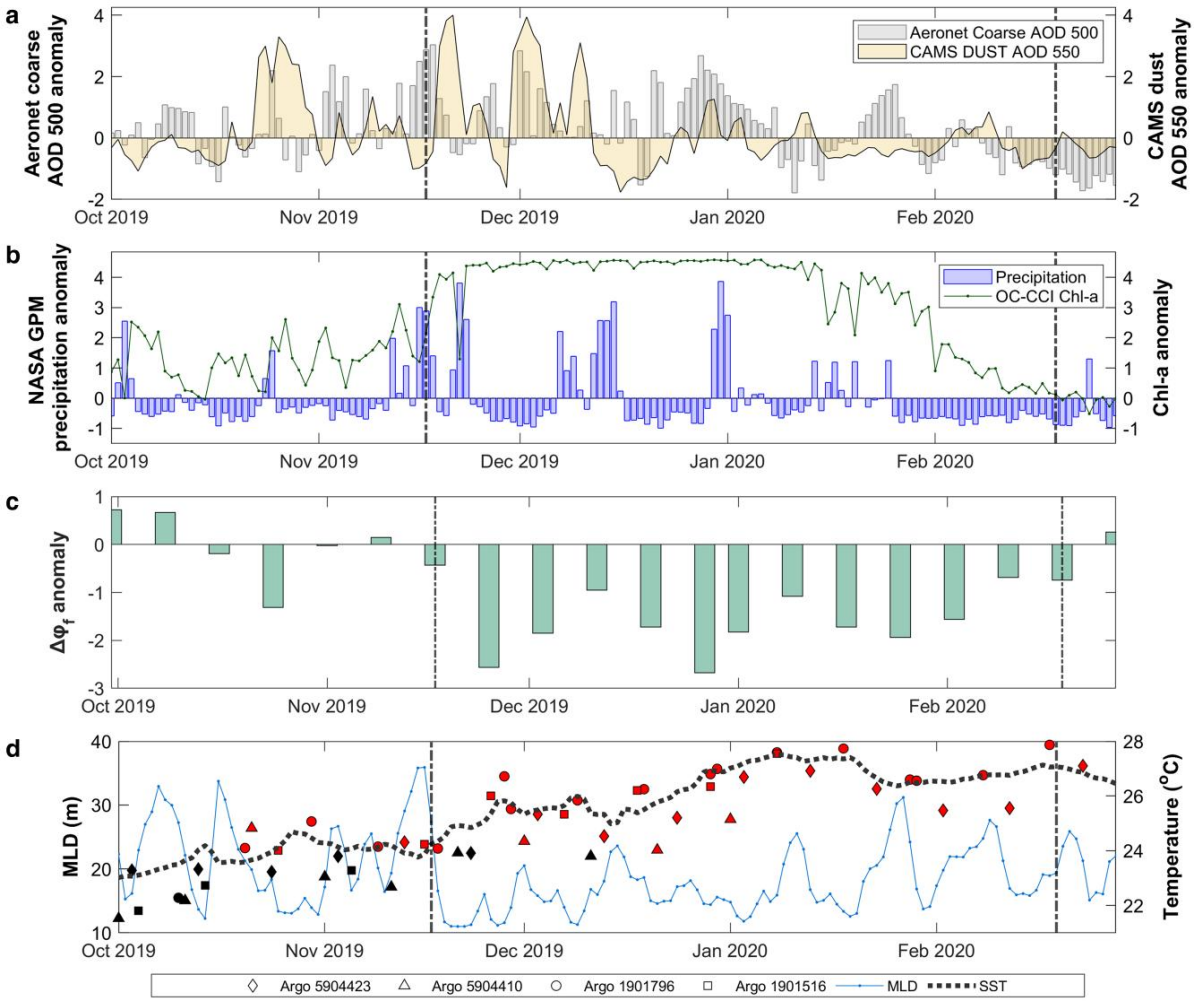
Temporal evolution of dust AOD, precipitation, iron stress and ocean physics during the austral spring and summer of 2019/2020. Standardized daily anomalies of a) Coarse mode AOD at 500 nm (acquired from the AERONET station at Réunion Island, Saint Denis [20.901°S, 55.485°E]) and Dust AOD at 550 nm (CAMS-ECMWF reanalysis) b) Precipitation (NASA GPM Mission) and Chl-a concentration (OC-CCI v6.0) c) 8-day chlorophyll fluorescence quantum yield standardized anomalies (a proxy for iron stress) computed from MODIS R2022 data following equation A8 in Behrenfeld et al. (41) d) (MLD, Mercator GLORYS Ocean Reanalysis) with Argo-derived mixed layer temperatures. Time series are based on the area-averaged variables over the defined Madagascar bloom region (see Fig. 1a). Daily anomalies of CAMS Dust AOD and precipitation were computed relative to the period 2003 January–2020 December, whereas anomalies of Chl-a, SST and MLD were computed relative to the period 1998 January–2020 December. The anomalies of coarse AOD from AERONET were computed relative to the period 2009 January–2020 December. The black diamond, circle, triangle, and square symbols represent mixed layer temperatures, acquired from the four Argo floats (Fig. S12), that was below 24 °C—the lower limit of the ideal temperature range for the growth of nitrogen fixers, such as *Trichodesmium* (46). The equivalent red markers represented mixed layer temperatures above 24 °C (diamond, circle, triangle, and square symbols represent Argo WMO 5904423, 1901796, 5904410, 1901516, respectively). The dotted, black line represents SST (OSTIA) spatially averaged within the northwest area of the bloom region (Fig. S13), which marks the initiation of the bloom as two mesoscale eddies (Fig. 1a).

**Fig. 3.**
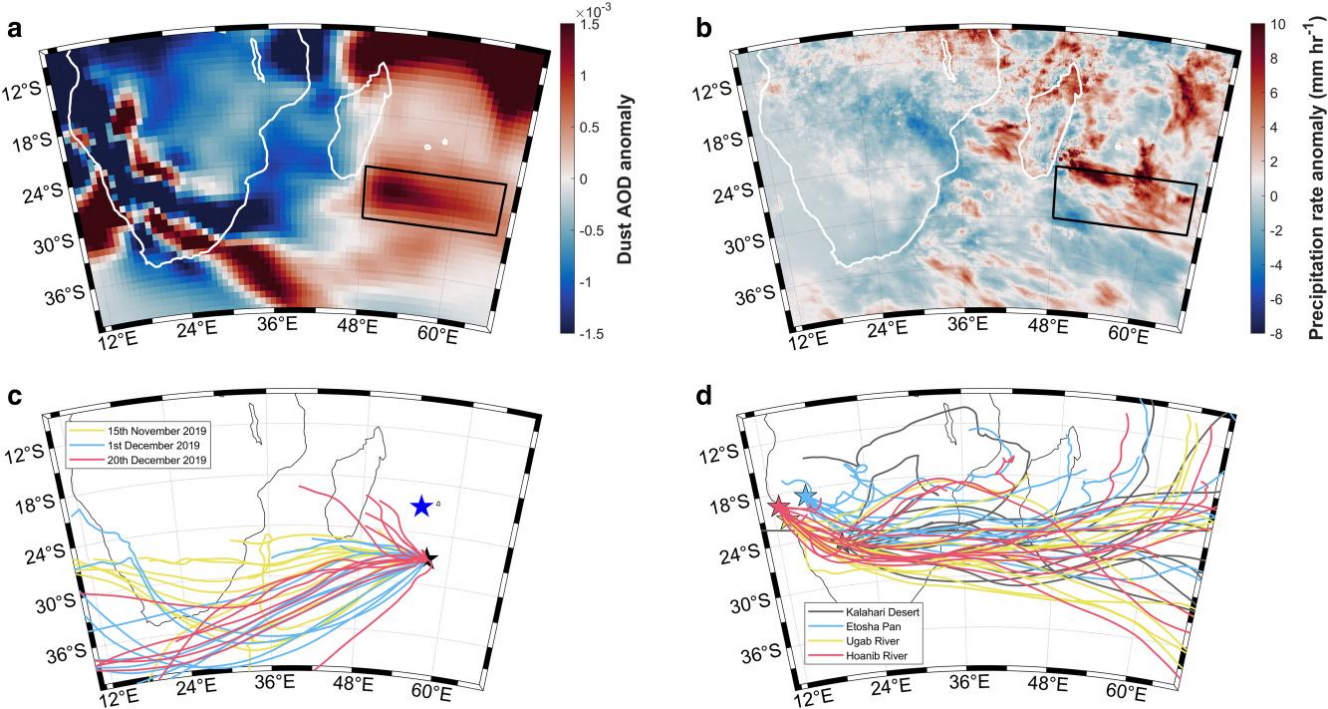
Transport and deposition of atmospheric dust aerosols over the bloom region. a) Spatial composite of CAMS dust AOD daily anomalies averaged over the period 2019 November15th–December 31st. This period was selected to encompass the whole period of increasing Chl-a concentration detected within the bloom region (the daily progression of the dust AOD signal from sources in Southern Africa toward southeast Madagascar is presented in Supplementary Movie 1) b) Spatial composite of daily precipitation rate anomalies (NASA GPM) averaged over the equivalent period c) HYSPLIT 7-day backward air parcel trajectories released for three separate days during the main phytoplankton growth period: November 15th, December 1st and December 20th. Air parcels released from the center of the bloom area (27°S, 57°E, black star in Fig. 3c) support the origin of nutrient-rich aerosols from key dust sources areas identified within the dryland regions of Southern Africa. The blue star highlights the location of the AERONET station at Réunion Island, Saint Denis d) HYSPLIT 14-day forward trajectories released from four potential dust-source areas in Southern Africa on 2019 November 10th. Key potential dust sources areas were selected based on previously identified dust sources within Southern Africa and include the Kalahari Desert (25°S, 20°E), Etosha Pan (18.80°S, 16.30°E), as well as the Hoanib (19.48°S, 12.76°E) and Ugab (21.18°S, 13.60°E) river valleys situated along the Namibian Skeleton Coast (16, 47, 48).

We explored atmospheric deposition of dust as an alternative mechanism of phytoplankton fertilization in the South Indian Ocean (45). To highlight the temporal evolution of the bloom and its potential drivers, we present standardized anomalies of dust aerosol optical depth (AOD) (CAMS reanalysis) over the bloom region (Figs. 1a and 2), and in situ coarse mode AOD retrieved by an AERONET station situated on Réunion Island, Saint Denis—the closest aerosol sampling station to the bloom region (blue star in Fig. 3c). As the mass of dust particles is predominantly comprised of the coarse mode (49), we opted to use this parameter as an independent, in situ index of atmospheric dust aerosols over the broader Madagascar region.

Coinciding with the bloom initiation in 2019 mid-November, CAMS dust AOD and in situ coarse mode AOD increased significantly and rapidly, reaching 3–4 SD above daily climatological values (Fig. 2a). In fact, dust AOD anomalies averaged over the bloom region were the highest observed over the entire 17-year CAMS time series for the November–December period (Figs. 3a and S14). Abrupt declines in dust and coarse mode AOD co-occurred with consecutive days of anomalously high precipitation (≥3 SD higher than respective daily climatological averages, Fig. 2b, purple-shaded bars), indicating increased dust wet deposition. The subsequent rapid increase in Chl-a to unprecedented concentrations (4 SD higher than respective daily climatological averages, Fig. 2b, green line) highlights the effect of these atmospheric deposition events on phytoplankton production.

Supporting a sudden relief from iron stress via dust deposition, we detected a strong negative anomaly (∼2.7 SD) in the chlorophyll fluorescence quantum yield (Δφ_f_) in 2019 mid-November, coinciding with the initial aerosol-deposition event and the start of the bloom (Fig. 2c). Further increases in dust AOD over the bloom region, as well the in situ coarse mode at Reunion Island, occurred in early- and late-December, followed by additional heavy, prolonged rainfall events that sustained high Chl-a concentrations and reduced iron stress (Figs. 2b, c). The subsequent decrease in Chl-a in mid-January was paralleled by an extended period of precipitation and dust/coarse mode AOD anomalies that were negative, or close to climatological values, until the bloom terminated in 2020 late-February.

Temperature is known to be a constraining factor on the development of nitrogen-fixing phytoplankton (46, 50–53). A common thermal optimum of ∼25 °C for biological nitrogen fixation has been identified across terrestrial and marine ecosystems, which is most likely associated with the temperature dependency of the nitrogenase enzyme that remains ubiquitous across taxa (53). To investigate the potential role of stratification and seasonal warming in bloom development, we analyzed the mixed layer depth (MLD), satellite-derived sea surface temperatures (SSTs) and in situ, Argo-based estimates of the average temperature within the mixed layer (Fig. 2d). Consistent with the typical onset of warmer, stratified conditions in austral summer (29–31), the MLD between October and early-November was shallow and fluctuated between 20 and 35 m (Fig. 2d). Despite earlier observations of significantly high AOD values coupled to a heavy but very short (∼1 day) precipitation event in October (Fig. 2b), only when temperatures increased, and consistently remained at ∼24–25 °C, did the bloom initiate and propagate (Fig. 2d). Further investigation of temperature limitation revealed that colder SSTs (<24 °C) occurred within the northwest region of bloom area, prior to the initiation in mid-November (Figs. 2d and S13). This is spatially consistent with the location of maximum Chl-a concentrations (>0.8 mg m^−3^), which remained north of the position of the 24 °C isotherm (Fig. S13).

Between 2019 November and December, strong, positive dust AOD anomalies were present over parts of Namibia, Botswana, and western South Africa (Fig. 3a, and as evidently shown in Supplementary Movie 1). The dust AOD composite anomaly from 2019 November 15th to December 31st (Fig. 3a) shows that dust emissions occurred from northern Namibia, Botswana, as well as the Kalahari and Namib deserts. The remobilization of dune fields between November and January in the Southwestern Kalahari Pan Belt has been shown to activate dust emissions that are comparable in strength to other sources in Southern Africa (16, 47, 54, 55), whilst the Namib desert hosts a range of potential emission sources (pans, ephemeral rivers, and wetlands (16, 47)). Ephemeral river valleys of the Namib Desert contain fine grain sediment that may have ∼43 times greater concentrations of bioavailable iron relative to other active dust sources in Namibia and Botswana (8). Analyses on the composition of mineral dust from gravel plans in the coastal Namibian desert have further quantified the soluble iron content in aerosol dust missions and its potential implications for ocean biogeochemistry (56). Ultimately, these results demonstrate that multiple potential sources of iron-rich dust aerosols over Southern Africa were active during the bloom period.

From 2019 mid-November to December, daily anomalies of precipitation rate over the bloom region and Mozambique Channel were high (>10 mm h^−1^) and can be easily distinguished from lower rates of precipitation in the adjacent regions of the Southwest Indian Ocean (Fig. 3b). Strong precipitation events, which contribute to the scavenging of aerosols from the atmosphere via wet deposition (57), are spatially consistent with the areas of increasing Chl-a concentration that marked the initiation and development of the bloom (Figs. 1a to c). The equivalent composite anomaly of dust wet deposition (Fig. S15), based on model reanalysis outputs from MERRA-2, is congruent with satellite-based observations of the precipitation anomaly (Fig. 3b). Furthermore, dust wet deposition anomalies within sub-areas of the bloom region were unprecedented, and exceeded 4.5 SD above the climatological mean on certain days (Fig. S15).

Backward and forward air parcel trajectories further corroborate the deposition of dust aerosols as the predominant driver of the 2019/2020 Madagascar bloom (Figs. 3c, d). The 7-day backward trajectories, released from the center of the Madagascar bloom area during three separate weeks in November and December, highlight the clear eastward transport from Southern Africa toward southeast Madagascar waters (Fig. 3c). Similarly, 14-day forward air parcel trajectories, released on November 10th from previously documented Southern African dust-source areas (16, 47, 48), collectively demonstrate consistent eastward dust aerosol transport toward Madagascar, ultimately reaching the bloom region approximately within the same week that the bloom initiated (∼2019 November 17–24th, Figs. 2b and 3d).

**Fig. 4.**
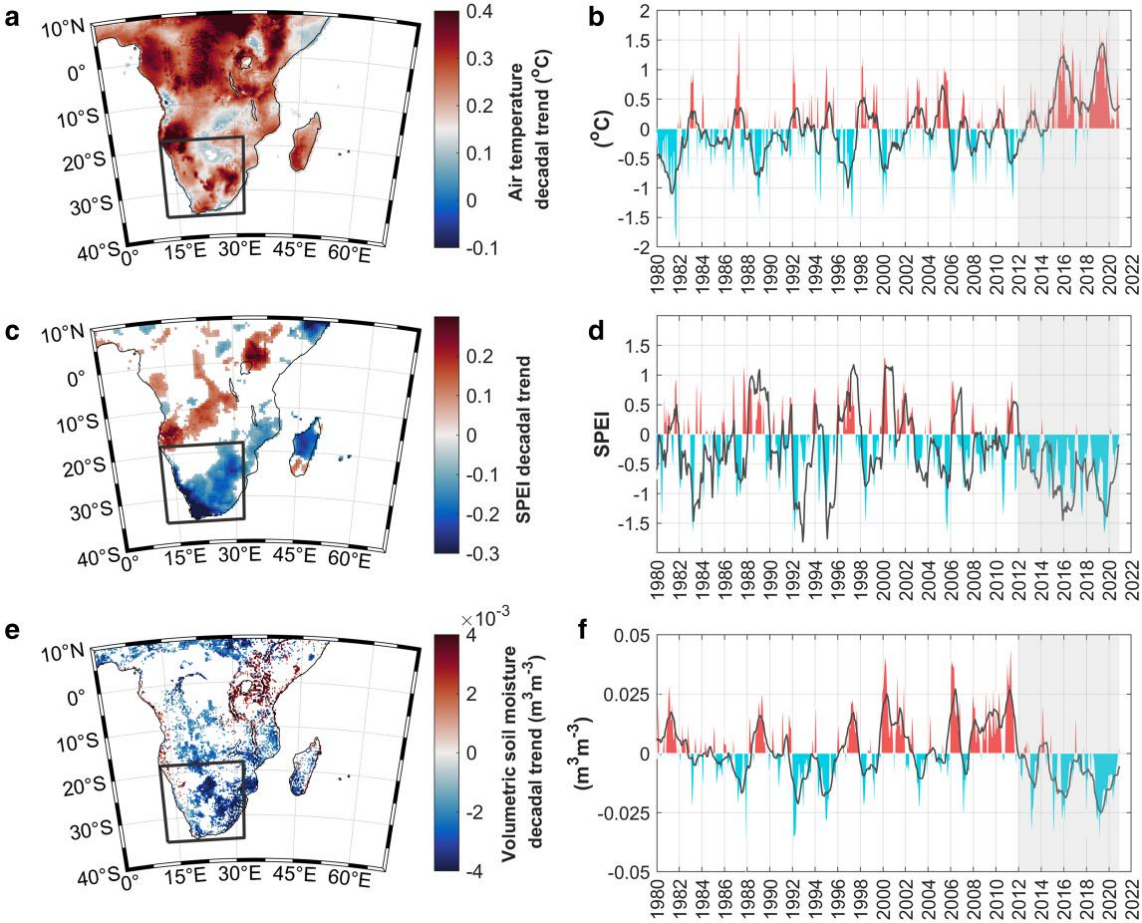
Southern Africa experienced a prolonged drought during the last decade. Spatial maps representing, respectively, the decadal trends over Southern Africa in a) air temperature (ERA5 ECMWF), c) the SPEI, a commonly used drought index that has been specifically produced for monitoring the effect of climate change on drought severity (59, 60) and e) volumetric soil moisture (ESA SM-CCI). All trends were computed over the period 1980–2020. Pixels characterized by a *P*-value ≥ 0.05 have been masked. Corresponding monthly time series of anomalies for b) air temperature, d) SPEI and f) volumetric soil moisture, averaged over Southern Africa (35–18°S; 12–32°E, represented by the black box in the maps). Anomalies were computed relative to the period 1980 January–2020 December. The gray shaded areas represent the period 2012–2020, which was characterized by positive air temperature anomalies, ongoing drought, and negative soil moisture anomalies. Note that the decade spanning 2011–2020 has been documented as the warmest on record with respect to the global land surface temperature anomaly (61). The dark gray lines in b) and f) show the 12-month moving mean whilst in d) it represents the SPEI acquired at a 12-month timescale. Negative SPEI values are indicative of drought events.

What factors may have driven emissions from Southern Africa that eventually stimulated the 2019 Madagascar bloom? Dust emission, transport and deposition are regulated by climate (58). Since 1980, air temperatures over broader Southern Africa have exhibited a significant, increasing trend, paralleled by stronger drought conditions (as indicated by the negative trends in the SPEI drought index) and soil moisture (Fig. 4). The most striking changes in these parameters occurred from 2012 to 2020, a period characterized by consistently high air temperature anomalies and continual drought (Fig. 4b, d, f). Prolonged episodes of drought reduce soil moisture (62), and consequently, vegetation cover. This, in turn, can lower the threshold wind-friction velocity required to mobilize soil particles and subsequently enhance dust emissions (63, 64). Supporting this, we detected a strong, significant negative annual trend in the Normalized Difference Vegetation Index (NDVI, Fig. S16). The contribution of dust aerosols from Southern Africa over the last century has reportedly doubled due to a combination of drier climate conditions and increasing anthropogenic activities (65). Additionally, recent analyses on the long-term wind erosion risk over Southern Africa have demonstrated that the Namib and Kalahari Deserts, as well as western parts of South Africa, are medium–high risk areas susceptible to wind erosion and more frequent dust storms (66).

Once dust aerosols become airborne (e.g. via direct aerodynamic lifting or saltation processes), they can be transported for thousands of kilometers (67). Long-range aerosol transport is predominantly determined by meteorological conditions and regional atmospheric circulation patterns (68). Previous analyses of tropospheric atmospheric trajectories over Southern Africa have revealed that the mean circulation field over the subcontinent is dominated by subtropical anticyclonic conditions (68), which both influence the wet/dry conditions over Southern Africa (69), and drive the easterly or westerly transport of aerosols to the Indian and Atlantic Oceans, respectively.

Extreme climate events and alterations to weather patterns are controlled by large-scale climate oscillations (70–72). Dominant controls of tropospheric variability in the SH include the El Niño-Southern Oscillation (ENSO), the Southern Annular Mode (SAM) (73), and the Indian Ocean Dipole (IOD). Although a neutral ENSO period, 2019 November–December coincided with one of the most negative phases of the SAM observed over the last 40 years (74). During negative SAM phases in the SH, the westerly wind belt around Antarctica expands equatorward (75). Associated cold fronts, low-pressure systems and intensification in regional winds may have been contributing factors toward the unusually strong episodes of dust aerosol transport and rainfall over Southern Africa and Southeast Madagascar, respectively. Long-term linkages between equatorward shifts in the westerly wind belt and enhanced dust transport from Southern Africa have been investigated during the Holocene (65) and generally align with the analyses presented here. However, broader climate processes governing dust emission, transport and subsequent deposition are complex and potentially antagonistic, and ultimately warrant further investigation. The austral spring of 2019 was also influenced by the strongest positive IOD in four decades (71, 72), contributing to the 2019 Australian megafires (27, 76) and droughts over multiple Indian Ocean rim countries, including Southern Africa (71). The frequency of extreme positive IOD events, which can bring severe drought to Indian Ocean rim countries, is projected to intensify in response to higher greenhouse gas emissions (77, 78).

Based on the satellite ocean color record, there is no doubt that this bloom was anomalous. Detailed, step-by-step analyses on alternative physical mechanisms that may have enhanced nutrient supply, including vertical mixing, Lagrangian transport, and light availability are presented in the Supplementary material. These analyses collectively indicate that the role of such processes was minimal, and this exceptional phytoplankton bloom most likely resulted from nutrient stress relief via atmospheric dust deposition. However, we acknowledge that causal attribution is challenging with natural events and that our study contains some inherent limitations. First, due to the scarcity of in situ data in the region during this event, it was not possible to provide a direct ground truth validation of ocean fertilization. Considering the increased potential for future dust deposition events, we emphasize the importance of directed in situ data collection campaigns to identify nutrient limitation regimes in the broader region. We also recognize the value of alternative methods, such as model simulations, which would allow focused hypothesis testing and the isolation of interactions between variables within the natural system. We recommend such an approach as a continuation of this work.

As global climate change intensifies over the 21st century (1), Earth system models predict declines in oceanic primary production, albeit with large uncertainties (79–82). Future alterations to primary productivity may perturb the ocean biological carbon pump, a key mechanism that ultimately modulates atmospheric CO_2_ concentrations (83). Although previously characterized as a region where air-sea CO_2_ fluxes are near equilibrium, in 2019/2020 the Madagascar bloom was a strong CO_2_ sink (30). Since atmospheric aerosol-deposition stimulates considerable biological responses over the global ocean (43) and global dust loadings have increased (84), in the future, ocean CO_2_ uptake by phytoplankton blooms could be enhanced by more frequent extreme aerosol-deposition events (e.g. droughts and wildfires (27)) driven by climate change. If we are to forecast the evolving functional role of oceans in a warmer Earth, it is necessary to improve our understanding of the interlinked negative feedback loop involving land, atmosphere, and ocean processes.

## Materials and methods

### Satellite ocean color data

Version 6.0 of the European Space Agency's Ocean Colour Climate Change Initiative (ESA OC-CCI) was used in this study (85). The OC-CCI product consists of merged and bias-corrected Chl-a data obtained from the Sea-Viewing Wide Field-of-View Sensor (SeaWiFS), Moderate Resolution Imaging Spectroradiometer (Aqua-MODIS), Medium Resolution Imaging Spectrometer (MERIS), Visible Infrared Imaging Radiometer Suite (VIIRS), and Sentinel3A-OLCI satellite sensors. Level 3, daily and 8-day mapped Chl-a data were acquired at a spatial resolution of 4 km from http://www.esa-oceancolour-cci.org, spanning a 24-year period from 1998 to 2020. We note that changes in satellite coverage can impart variability into spatio-temporally averaged ocean color records. Therefore, to ensure our results were not impacted by fluctuations in satellite coverage, we assessed the spatial coverage of Chl-a observations from the OC-CCI dataset during the austral spring/summer of 2019/2020 (Fig. S17). During the 2019/2020 bloom, the number of valid pixels ranged between 80 and 100%, except for a small decrease in coverage in early-December (60%). Overall, we believe that the data coverage provided by the OC-CCI product is adequate for reliably conducting a satellite-based analysis of the 2019/2020 Madagascar bloom. We refer the reader to the OC-CCI v6.0 Product User Guide at https://climate.esa.int/en/projects/ocean-colour/key-documents/ for a more extensive overview of processing, sensor merging, and uncertainty quantification.

### Computation of phytoplankton phenology metrics

We note that spatial averages were computed within the geographical limits defined in previous literature on the South–East Madagascar Bloom (28, 29). To illustrate the unprecedented scale of the bloom and eliminate any potential bias from choosing a specific study area, we performed an iterative analysis on 3,750 geographical boxes, each 5 × 5°, within the broader waters around Madagascar and West Africa (15°S–40°S, 30°E–80°E, Fig. S18). Starting from the edge of this broader domain, the geographical box was moved iteratively 1° eastward and 1° southward, and the Chl-a monthly time series was computed. These analyses confirm that, regardless of the defined study region, Chl-a during the austral summer of 2019/2020 reached unprecedented values. Aside from the 2019/2020 event, previous blooms were identified in the austral summers of 1999, 2000, 2002, 2004, 2006, 2008, 2009, 2012, 2013, and 2014, following *Dilmahamod* et al (29). (Their Fig. 2). Two additional recent blooms were visually identified in 2017 and 2018, based on the Chl-a monthly anomaly (Fig. 1d). To quantify the precise timing (in weeks) of bloom initiation and termination we utilized the cumulative sums of anomalies method, based on a threshold criterion, to estimate phytoplankton phenology metrics (bloom initiation, termination, and duration) during bloom years. The threshold criterion method is centered on the concept that the occurrence of a phytoplankton bloom corresponds to a significant increase in Chl-a above “normal” concentrations (86–88). The cumulative sum of anomalies method requires a gap-free Chl-a time-series as an input, otherwise phenology metrics cannot be calculated. Hence, to improve the coverage of Chl-a satellite data, we applied a linear interpolation method that fills gaps in the time series. The interpolation method is based on the MATLAB subroutine *inpaint_nans*, which interpolates missing data using a linear least squares approach (89). We defined the threshold criterion as the long-term median of the entire Chl-a time series, plus 20%. This threshold was selected as it was found to be the most representative of the austral summer bloom initiation and termination over the 24 years. We note that various thresholds (5, 10, and 15%) have been utilized in the global oceans, depending on the type of analysis (e.g. interannual or climatological) The 8-day Chl-a data, spatially averaged over the bloom area, were isolated for the period spanning 1997 August 29–2020 August 20. Using this threshold, Chl-a anomalies were computed by subtracting the threshold criterion from the 8-day time series. The cumulative sums of anomalies were then calculated for each of the defined bloom years. Increasing (decreasing) trends in the cumulative sums of anomalies represent periods when Chl-a concentrations are above (below) the threshold criterion. The gradient of the cumulative sums of anomalies was then used to identify the timing of the transition between increasing and decreasing trends. The initiation of the phytoplankton bloom corresponded to the 8-day period when Chl-a concentrations first rose above the threshold criterion (i.e. when the gradient of the time series first changed sign). The termination of the phytoplankton bloom was computed as the time when the gradient first changed sign following the occurrence of the maximum Chl-a concentration in the time series (the growth peak). The total duration of the phytoplankton growth period was calculated as the number of 8-day periods between the timings of initiation and termination. As some bloom years experienced a secondary phytoplankton growth period during austral winter, the phenology algorithm was adjusted to detect fluctuations above/below the threshold criterion between October and May for each bloom year, thus enabling us to isolate austral spring and summer.

### Satellite-derived primary production and export production

We acquired monthly estimates of phytoplankton primary production and export production from the ESA Biological Pump and Carbon Exchange Processes project (BICEP, https://bicep-project.org/). Primary production was modeled using ocean color products and a spectrally resolved primary production model (90, 91). This model integrates the vertical structure of phytoplankton, acquired from a large database of in situ Chl-a profiles, and simulates changes in photosynthesis as a function of irradiance using a two-parameter photosynthesis verses irradiance function (90). Photosynthesis vs. irradiance (P-I) parameters were acquired from a global database of in situ measurements (90). PAR products were obtained from the National Aeronautics and Space Administration (NASA). Export production was defined as the steady-state Net Community Production (NCP), with temporal lags accounted for, and a well-defined depth horizon, from which community production is integrated over (92). The estimates of export production utilized in this study are based on the NCP algorithms presented in Li & Cassar (93). We refer the reader to the related documents section at https://catalogue.ceda.ac.uk/uuid/a6fc730d88fd4935b59d64903715d891 for further information on the algorithms used for the computation of export production. Datasets of primary production (94) and export production (92) have a horizontal resolution of 9 km and were available for the periods 1998 January–2020 December and 1998 January–2019 December, respectively.

### Chlorophyll fluorescence quantum yield

Level 3 global fields were acquired by the MODIS instrument onboard the Aqua spacecraft for the period 2003–2020 January (https://oceancolor.gsfc.nasa.gov/data/overview/). Specific products were acquired at a ∼9.25 km spatial resolution and 8-day temporal resolution, and included Chl-a concentration, the instantaneous broadband irradiance (iPAR, uEin m^−2^ s^−1^), the daily-integrated broadband irradiance (PAR, Ein m^−2^ d^−1^), and Chlorophyll Fluorescence Line Height (nFLH, W m^−2^ μm^−1^ sr^−1^). The products of nFLH, iPAR, PAR, and Chl-a were subsequently combined to estimate the chlorophyll fluorescence quantum yield (φf, dimensionless) following Behrenfeld et al. (41).

### EKE and polarity

We calculated the EKE as it is directly proportional to eddy diffusivity (29, 95), which is known to impact the dispersion of Madagascar bloom (29, 35). The EKE was computed as follows:


$$\mathrm{EKE}=\frac{1}{2}\;\sqrt{u^{2}+v^{2}}$$

where *u* and *v* are the zonal and meridional components of surface currents, respectively. We calculated the EKE climatological seasonal cycle (for 1997 September–2020 December) and Decembers EKE from 1997 to 2020 over the bloom area. We also retrieved the number of cyclonic versus anticyclonic eddies occurring within the bloom area (Fig. 1a) for 1997–2020 Decembers, using the output of an eddy detection algorithm based on Sea Level Anomaly (SLA) and streamlines (approximated by SLA contours under the geostrophic assumption (96)). This approach has been commonly used for identifying mesoscale eddies in ocean regions deeper than 200 m (96–101). The eddy detection algorithm is based on the MATLAB subroutine *SimpleEddyDetection.m* (102). The algorithm identifies eddies by finding their center and edges (96, 100). An eddy centre is found by the mass centre of the innermost closed SLA contour. Then, the closed contours surrounding the eddy centre are identified as their SLA values change monotonously outward from the centre. The eddy edge is the outermost closed SLA contour (96). The eddies-identifying criteria are adapted from *Xu* et al (100). and *Zhang* et al (96). Surface currents and SLA fields used to derive the EKE and eddies polarity for 1997–2021 were obtained from the satellite altimetry derived SLA and absolute geostrophic *u* and *v* processed by the Collecte Localisation Satellites (previously by AVISO [Archiving, Validation and Interpretation of Satellite Oceanographic Data]) and distributed by the Copernicus Marine Environment Monitoring Service (CMEMS, http://marine.copernicus.eu/services-portfolio/access-to-products/). These multisatellite Level-4 products are available daily at 25 km spatial resolution for the period 1993–2021 from the delayed time DUACS_DT2018 version. Satellite altimetry data have known limitations such as sensor land contamination near the coast (103). However, the study area is mainly composed of offshore waters. Furthermore, the product version used here shows an error reduction by more than 15% in geostrophic currents estimation in coastal zones (104).

### Aerosol analysis

Datasets of dust AOD were acquired from the Copernicus Atmosphere Monitoring Service (CAMS; http://atmosphere.copernicus.eu), which is part of the European Earth-observation programme Copernicus (https://www.copernicus.eu/en) produced by the European Centre for Medium-Range Weather Forecasts (ECMWF). CAMS provide global reanalysis datasets of greenhouse gases, reactive trace gases, aerosol concentrations as well as several meteorological variables (105). The CAMS reanalysis consists of three-dimensional time-consistent atmospheric composition fields available at a frequency of 3–6 h, from 2003 to 2020. For this study, 3-hourly fields were averaged into a daily dataset. The CAMS aerosol model component is based on the Integrated Forecasting System meteorological model (106) and contains, amongst other parameters, 3 prognostic tracers for dust aerosols (105). CAMS aerosols are assimilated with MODIS satellite observations (107) of total AOD at 550 nm. Long-term, continuous measurements of aerosol optical properties were acquired from the Aerosol Robotic Network (AERONET) website (https://aeronet.gsfc.nasa.gov) at the “REUNION_ST_DENIS” site (20.901°S, 55.485°E), for the period 2009 January–2020 December. Specifically, we retrieved Level 2.0, daily observations of the coarse mode of AOD at 500 nm, generated using the Spectral Deconvolution Algorithm (108, 109). For the computation of the coarse mode AOD climatology and respective standardized anomalies (Fig. 2), a linear interpolation scheme (MATLAB function *interp1*) was applied to fill gaps in the time series. We note that were no gaps in the raw AERONET data during the coincident periods of enhanced AOD and precipitation in 2019 mid-November and early-December, when the bloom developed rapidly.

### Precipitation rate

We acquired measurements of precipitation rate from the Global Precipitation Measurement (GPM, https://gpm.nasa.gov), a joint mission of the NASA, and Japan Aerospace Exploration Agency (JAXA). We acquired precipitation rates from the recommended IMERG Final Run algorithm, which merges, intercalibrates, and interpolates satellite microwave precipitation estimates, microwave-calibrated infrared (IR) satellite estimates, precipitation gauge analyses, and other potential precipitation estimators during the TRMM and GPM eras over the entire globe https://disc.gsfc.nasa.gov/datasets/GPM_3IMERGDF_06/summary?keywords=%22IMERG%20final%22. Daily observations of precipitation rate are available at a spatial resolution of 0.1 × 0.1° and were acquired over the bloom area between 2003 January and 2020 December.

### Dust aerosol wet deposition

Estimates of total dust aerosol wet deposition fluxes used in this work were acquired from the Modern-Era Retrospective analysis for Research and Applications, Version 2 (MERRA-2, https://gmao.gsfc.nasa.gov/reanalysis/MERRA-2/). MERRA-2 is the latest version of global atmospheric reanalysis for the satellite era produced by NASA Global Modeling and Assimilation Office (GMAO) using the Goddard Earth Observing System Model (GEOS) version 5.12.4. Hourly data of wet deposition were acquired over the period 1998 January–2020 December and were averaged into daily composites. Dust aerosols are represented with five bins that correspond to dry size ranges (*µ*) and densities (kg m^−3^). For this study, we computed the total wet deposition by summing the wet deposition fluxes of the five size bins.

### Air temperature

Monthly observations of ERA-5 air temperature at 2 m above the land surface were acquired from the Copernicus Climate Change Service (C3S) Climate Data Store (https://cds.climate.copernicus.eu/#!/search?text=ERA5&type=dataset), for the period 1980 January–2020 December. Data have a horizontal resolution of 0.25° × 0.25°.

### Atmospheric trajectory analysis

Forward and backward trajectories were respectively used to track the transport and sources of aerosols in the atmosphere via the Hybrid Single-Particle Lagrangian Integrated Trajectory model (HYSPLIT) (110). Meteorological data were acquired from NCEP/NCAR Reanalysis (111) spanning the period from November to December 2019. For back trajectories, we traced the origins of aerosols that were transported to the bloom region during three separate 7-day periods within the broader period spanning 2019 November 15th–2019 December 31st: November 15th, December 1st and December 20th. The model was initiated at the center of the bloom region (27°S, 57°E, 5,500 m above sea level) to calculate back trajectories of 168 h (7 days), with a trajectory launched every 12 h. Forward trajectories of 336 h (14 days) were launched every 24 h from four potential dust-source areas in Southern Africa on 2019 November 10th. Key potential dust sources areas were selected based on previously identified dust sources within Southern Africa and include the Kalahari Desert (25°S, 20°E), Etosha Pan (18.80°S, 16.30°E), as well as the Hoanib (19.48°S, 12.76°E) and Ugab (21.18°S, 13.60°E) river valleys situated along the Namibian Skeleton Coast (16, 47, 48).

### Mixed layer depth

Daily outputs of MLD over the bloom area were acquired from the GLORYS12V1 ocean reanalysis provided by the CMEMS (https://doi.org/10.48670/moi-00021), at a horizontal resolution of 1/12° for the period 1998 January–2020 December. The model component of GLORYS12V1 is the Nucleus for European Modelling of the Ocean (NEMO) platform, driven at the surface by ECMWF ERA-Interim and ERA5 reanalyses for recent years.

### Sea Surface Temperature

For the computation of daily time series of SST, we used the Operational SST and Sea Ice Analysis (OSTIA) system, which provides global, daily averaged fields of SST at a 1/20° horizontal resolution (112), for the period 1998 January–2020 December (https://ghrsst-pp.metoffice.gov.uk/ostia-website/index.html). OSTIA uses a combination of satellite data from microwave and IR satellite instruments provided by the Group for High Resolution SST (GHRSST), along with in situ observations from the International Comprehensive Ocean-Atmosphere Data Set (ICOADS) database. OSTIA products have been validated by inter-comparisons with other historical datasets and are continuously validated with in situ measurements.

### Standardized precipitation evapotranspiration Index

SPEI dataset provides long-term, global information on drought conditions (59, 60). The SPEI is an improved drought index specifically suited for studies aimed at understanding the impacts of global warming on drought severity (60). Like other popular drought indices (113), the SPEI incorporates the effect of precipitation and potential evapotranspiration on drought severity. However, as drought may be driven by several processes operating at different timescales (114), the SPEI has an advantage over other drought indices in the fact that it has a multiscalar character, enabling the identification of different drought types and impacts. The SPEI is available at timescales ranging from 1 to 48 months, the selection of which is ultimately dependent on the type of analysis. Due to the prolonged nature of drought over Southern Africa reported between 2019 October and December, we opted to use an SPEI timescale of 3 months to realistically capture drought onset, relief, and intensity. Data for the period 1980 January–2020 December were obtained from Global SPEI database (https://digital.csic.es/handle/10261/268088). We note that time series of the SPEI were produced at timescales ranging between 1 and 6 months and remained consistent with the current 3-month SPEI index.

### Volumetric soil moisture

Satellite-derived observations of volumetric soil moisture were acquired from version 7.1 of the ESA Soil Moisture Climate Change Initiative (ESA SM-CCI v07.1, https://www.esa-soilmoisture-cci.org/). The ESA SM-CCI product uses a merging algorithm to generate a quality-controlled, super collocated, long-term (1978–2021) soil moisture dataset based on retrievals from multiple satellite sensors. Merged datasets are available as active-microwave-based only (ACTIVE), passive-microwave-based only (PASSIVE), and a combined active–passive (COMBINED) product. Here, the COMBINED global dataset was acquired at a daily temporal resolution for the period 1980 January–2020 December and aggregated into monthly averages. The data have a spatial resolution of 0.25 × 0.25° and are provided in volumetric units (m^3^ m^−3^). We refer the reader to the Product User Guide (https://esa-soilmoisture-cci.org/node/119) for further information.

### Core-Argo float observations

We acquired data from four Core-Argo floats via the online data selection tool of the Euro-Argo European Research Infrastructure Consortium (ERIC) (https://dataselection.euro-argo.eu/). The four floats ((ⅰ) WMO ID: 5904423, https://www.ocean-ops.org/board/wa/InspectPtfModule?ref=5904423, (ⅱ) WMO ID: 1901796, https://www.ocean-ops.org/board/wa/InspectPtfModule? ref=1901796, (ⅲ) WMO ID: 1901516, https://www.ocean-ops.org/board/wa/InspectPtfModule?ref=1901516, (ⅳ) WMO ID: 5904410, https://www.ocean-ops.org/board/wa/InspectPtfModule?ref=5904410) had cycle times of ∼10 days, drifting depths at 1,000 m (except for #1901516 at 1,500 m), and maximum profile depths of 2,000 m. They all bore SEABIRD_SB41CP sensors for measuring salinity, temperature and pressure, along with an extra sensor (DRUCK_2900PSIA) for pressure. In all cases, we used the ascending profiles’ adjusted values for temperature, salinity, and pressure of “good quality” data (flag value = 1). Adjusted temperature data were used for the calculation of the MLD per float. For the Argo MLD determination required to provide estimates of the average temperature within the mixed layer, we used a temperature difference-based criterion with a threshold value of 0.2 °C (difference between the surface layer [10 m] and the deeper water layers) (115). Mixed layer temperatures were computed by averaging the ARGO temperature observations above the computed MLD. These ARGO data were collected and made freely available by the International Argo Program and the national programs that contribute to it (https://argo.ucsd.edu, https://www.ocean-ops.org). The Argo Program is part of the Global Ocean Observing System.

### Normalized Difference Vegetation Index

We acquired satellite-derived monthly composites of the NDVI from the Terra MODIS sensor. Specifically, we downloaded the MODIS VI (MOD13) product, which provides consistent spatial and temporal time series comparisons of global vegetation conditions (https://modis.gsfc.nasa.gov/data/dataprod/mod13.php). MODIS NDVI products are based on surface reflectances that are corrected for molecular scattering, ozone absorption and aerosols. Version 6, level 3 data were acquired at a 1 km spatial resolution over broader Southern Africa, for the period 2000 February–2020 December. Prior to analysis, a quality control procedure was applied by masking pixels that ranked below the “Use with confidence” pixel reliability criteria.

### Photosynthetically active radiation

Level 3, daily, mapped data of PAR were acquired from the Aqua-MODIS sensor at a horizontal resolution of 4 km (https://oceandata.sci.gsfc.nasa.gov/), for the period 2019 October–2020 March. For the computation of average PAR within the mixed layer, we first calculated the diffuse attenuation at 490 nm [K_d_(490)] following Equation 8, and K_d_(PAR) following Equation 9, in Morel et al. (116). Following this, we computed PAR averaged within the mixed layer based on Equation 11 of Brewin et al. (117).

## Supplementary Material

pgae386_Supplementary_Data

## Data Availability

All data are included in the manuscript's Materials and Methods and Supplementary material sections.
