# Supplementary material for: An exceptional phytoplankton bloom in the southeast Madagascar Sea driven by African dust deposition
Source: PNAS Nexus. 2024 Oct 1;3(10):pgae386. doi: 10.1093/pnasnexus/pgae386 (PMC11443548; doi:10.1093/pnasnexus/pgae386)
Supplement: pgae386_Supplementary_Data [file pgae386_supplementary_data.zip › PNASNEXUS-PNASNEXUS-2024-00568-TR-s02.docx]

**Supplementary Materials for**

**An exceptional phytoplankton bloom in the southeast Madagascar Sea driven by African dust deposition**

**Authors:** John A. Gittings^1^, Giorgio Dall’Olmo^2^, Weiyi Tang^3^, Joan Llort^4^, Fatma Jebri^5^, Eleni Livanou^1^, Francesco Nencioli^6^, Sofia Darmaraki^1^, Iason Theodorou^1^, Robert J. W. Brewin^7^, Meric Srokosz^5^, Nicolas Cassar^8*^, Dionysios E. Raitsos^1*^

**Affiliations:**
^1^Department of Biology, National and Kapodistrian University of Athens; 15784 Athens, Greece
 ^2^Sezione di Oceanografia, Istituto Nazionale di Oceanografia e Geofisica Sperimentale – OGS; Borgo Grotta Gigante, Trieste, 34010, Italy
^3^Department of Geosciences, Princeton University; Guyot Hall, Princeton, NJ 08544, United States of America
^4^Barcelona Supercomputing Center; Plaça d'Eusebi Güell, 1-3, Les Corts, 08034 Barcelona, Spain
^5^National Oceanography Centre; Southampton, SO14 3ZH, United Kingdom
^6^Collecte Localisation Satellites; 31520 Ramonville-Saint-Agne, France
^7^Centre for Geography and Environmental Science, Department of Earth and Environmental Science, Faculty of Environment, Science and Economy; University of Exeter, Cornwall, United Kingdom
^8^Division of Earth and Climate Sciences, Nicholas School of the Environment, Duke University; Durham, NC, United States of America
^*^Corresponding authors. Email: draitsos@biol.uoa.gr; Nicolas.Cassar@duke.edu

**Lagrangian trajectory analysis**

The goal of the Lagrangian analysis was to identify the contribution of the advection of nutrient-rich shelf waters to the initiation and development of the bloom. These can come from two sources: a) continental shelves from the South-Eastern African region; b) the East coast of Madagascar.

Lagrangian trajectories were reconstructed based on the Lagrangian Manifolds and Trajectories Analyser described in van Sebille et al. (*1*) and already used to support, among others, works in the NW Mediterranean (*2*), southern Indian Ocean (*3*) and the Southern Atlantic Ocean (*4*). The analysis used global multi-satellite gridded geostrophic velocities (1/4∘ resolution) from the SSALTO/DUACS all-sat-merged data set (SSALTO/DUACS User Handbook, 2016) freely distributed by the European Copernicus Marine Environment Monitoring Service (CMEMS; <http://marine.copernicus.eu/>). Particles were released within our region of focus (box between 48^°^ to 66^°^ East and 24^°^ to 30^°^ South) at a spatial resolution of 1/8^°^ (∼12 km at 30°S) and then advected backward for 90 days. The advection is performed with Runge-Kutta fourth-order scheme and a 6-hr time step, with the velocity field interpolated bilinearly in space and linearly in time.

Within the region of focus, we also identified bloom areas based on 8-day composite OC-CCI surface Chl-a concentrations described in the manuscript. Specifically, a bloom was defined as the area where Chl-a concentrations were higher than 1.5 times the average value within the box (~ 0.158 mg m^-3 in our case). Blooms were identified on November 1^st^, November 17^th^ and December 3^rd^ in 2019. The Lagrangian analysis described in the previous paragraph was performed for each of those days. Here, we will focus on the results from December 3^rd^, 2019, corresponding to the day when the Chl-a bloom started to expand beyond the two initial eddies (Fig. S3). To better contextualize the results obtained in 2019, the same analysis was also performed for 2018 (November 25^th^, December 3^rd^ and 11^th^) and 2017 (December 3^rd^ and 11^th^), when small, localised increases in Chl-a occurred east of the southernmost Madagascar tip, like the ones initially observed in November 2019. Results from December 11^th^, 2018 will be discussed at the end of the section.

To identify the contribution of nutrient-rich shelf waters from the continental shelves of South-Eastern Africa, we conservatively defined the shelf boundary as the 1000 m depth isobath. Thus, we associated nutrient-rich shelf waters with any trajectory who crossed such isobath. To identify the particles off the Eastern Madagascar shelf, we applied a different selection method, since using a conservative approach as the 1000 m isobath seemed to still underestimate the contribution from this region. As the East coast of Madagascar is characterized by the strong East Madagascar Current flowing southward along the continental slope (*5*, *6*)*,* we assumed that any water parcel within the current could potentially come from the slope and thus be nutrient enriched. Thus, we defined Eastern Madagascar shelf particles, any trajectory which came from the area of strong mean velocities along the eastern Madagascar coast (Fig. S4).

Based on those two criteria, we classified all backward Lagrangian trajectories according to their area of origin. Fig. S5 shows the results of the classification for 60-day trajectories. Similar analyses for 30- and 90-day trajectories showed analogous results.

Of 172 particles from the Madagascar shelf, 91 are found within the bloom region (Fig. S5, top). Of 155 particles from the southeastern shelves, 138 are within the bloom (Fig. S5, middle). Those particles come mostly south Madagascar shelf and from a region of shallow waters from the Madagascar Plateau immediately south of Madagascar at ~33^o^S. As also confirmed by the 90-day particles, there was no direct contribution from shelves of the Southeast African continent. The particles coming from the Madagascar and southeastern continental shelves are all found along the eastern and northern boundaries of the two eddies characterized by the initial bloom. None of those particles are found east of 55^o^E. Therefore, while nutrient-rich continental shelf water might have indeed at least partially contributed to the formation of the bloom within the two eddies, they cannot explain the initialization and development of the much larger bloom east of those features. Indeed, the bottom panel in Fig. S5, shows that of the 3549 particles within the bloom region on December 3^rd^, 3320 remained over open ocean (i.e., depth > 1000m) for the full 60 days before, indicating that they could have not been nutrient enriched over the continental shelf.

Fig. S6 summarizes the results of the Lagrangian analysis by showing the distribution of the particles within the December 3 bloom, 60-days before. Almost 60% of the particles remains within the area of focus (85% if the northern and southern boundary of the area are moved to 22.5 S and 33 S, respectively. Thus only 15% of the particles found within the bloom originated from the continental shelves around Madagascar.

During a non-bloom year in 2018, results from the Lagrangian analysis on 11^th^ December 2018, shows that shelf waters might have contributed to increased Chl-a concentrations East of Madagascar (Figs S7-S8). Analogous results are also found for 2017 (not shown). Furthermore, even if not detected as a bloom region by our threshold, the analysis shows that the wavy pattern of higher (with respect to their surroundings) Chl-a concentrations between 24 and 27^o^S and up to 60^o^E have also received a contribution from shelf waters (grey trajectories). However, neither 2017 nor 2018 showed the development of a bloom of the magnitude and extent as the one observed in 2019. These results reinforce our hypothesis that nutrient-rich shelf waters alone could not explain the observed 2019 bloom and that therefore nutrient enrichment due to dust deposition must have played a decisive role in its initiation and development.

**Potential nutrient inputs from vertical mixing**

The injection of nutrient rich waters from vertical mixing/upwelling could constitute an alternative/complementary mechanism for the development of this unprecedented phytoplankton bloom. An in-depth investigation of this mechanism is provided below. For these analyses, the E.U. Copernicus Marine Service Information products MULTIOBS_GLO_PHY_TSUV_3D_MYNRT_015_012 (https://doi.org/10.48670/moi-00052) and MULTIOBS_GLO_BIO_BGC_3D_REP_015_010 (https://doi.org/10.48670/moi-00046) for temperature, salinity and biological variables were used, respectively. Density was calculated from temperature, salinity and pressure (derived from depth) using the Python seawater package (version 3.3, (*7*–*9*))

Below, we provide panels of Chl-a concentration (green lines) and the backscattering coefficient (b­_bp_, a proxy for particulate organic matter, blue lines), plotted versus depth, as well as their respective climatological values (black lines) (Fig. S9a, b). We also provide similar plots, with Chl-a and b­_bp_ plotted against density (Fig. S9c, d). Note that each parameter represents a spatial average over the bloom area (48 – 66^o^E, 30 – 24^o^S). The depth and density profiles of Chl-a and b­_bp_ show that the 2019-2020 Madagascar bloom developed as a surface bloom and is constrained within the 0 – 60 m surface layer. These results agree with previous observations of past summer blooms in the area (*10*), albeit all of them occurring later compared to the 2019-2020 bloom. The co-occurrence of the subsurface Chl-a and b­_bp_ maxima with lower densities throughout the bloom period supports the hypothesis that the bloom occurred within warmer surface waters and was mainly seeded from nutrient inputs at the surface, as opposed to nutrients from the mixing of colder, nutrient-rich deeper layers.

Additionally, the vertical structure of the water column along a longitudinal transect (48-66^o^E, 27-27.5^o^S) crossing the middle of the bloom area was investigated during the bloom and compared to the previous year (November-February 2018/2019) when no bloom was observed (Fig. S10). Examining the longitudinal section under non-bloom conditions, from November 2018 to February 2019, the Chl-a maximum depth varies between 60-120 m along the longitudinal transect and closely follows the 25.5 kg m^-3^ isopycnal throughout the period of interest. Multiple eddies are present in the area causing significant vertical displacement of the isopycnals, along the transect. These vertical movements are not associated with any significant Chl-a increase.

During the 2019 Madagascar bloom (Nov 2019 - Feb 2020) the vertical movement of the isopycnals along the longitudinal transect can also be observed. Right before the bloom onset, the Chl-a maximum displays a typical behaviour and is closely coupled with the 25.5 kg m^-3^ isopycnal, located within the 60-120 m layer. From 2019-11-20 onwards the 25.5 kg m^-3^ isopycnal does not show any important vertical displacement towards the surface. Patches of enhanced Chl-a concentration start to appear along the longitudinal transect, associated with cyclonic eddies present in the area. The Chl-a maximum is located between 20 – 60 m and is now uncoupled with the isopycnal. The initiation of Chl-a maximum migration towards the surface is synchronized with the first anomalous wet deposition event, suggesting that micronutrients input to the surface waters from aeolian deposition was the main driver of the 2019-2020 summer bloom. As the surface-subsurface bloom further develops, the Chl-a maximum becomes completely uncoupled with the isopycnal, during the bloom peak period (2019-11-27 to 2020-1-22), especially within the western part of the bloom box (48^o^ - 60^o^ E). As the bloom decays the Chl-a maximum gradually retreats to deeper layers and once again follows the 25.5 kg m^-3^ isopycnal.


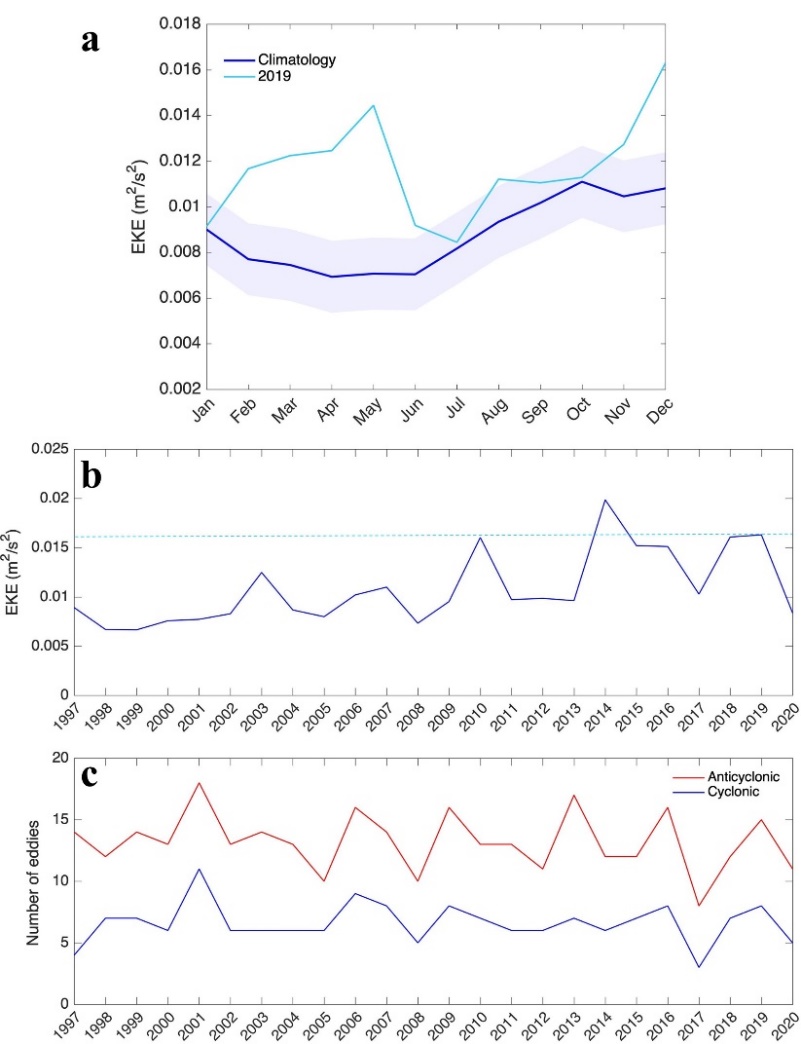

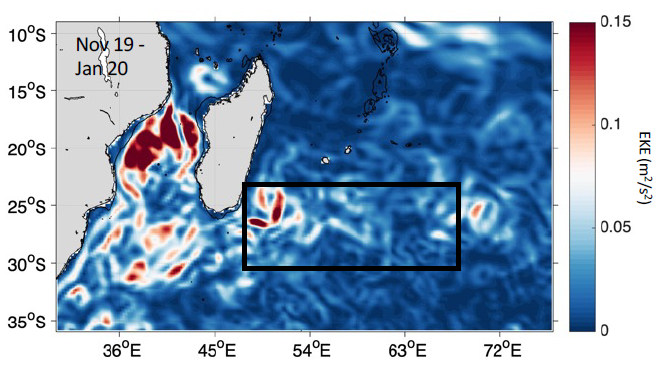


**d**

**Fig. S1. (a)** Seasonal climatology of Eddy Kinetic Energy (EKE) (dark blue line) plotted alongside the monthly time series of EKE for 2019 (turquoise line) in the bloom region. The blue shading represents +/- 1 monthly climatological standard deviation. The EKE reaches its seasonal peak between October and December, coinciding with the onset and propagation of the 2019/2020 Madagascar bloom. **(b)** Time series of December EKE from 1997 – 2020. The turquoise dashed line represents the EKE value reached in December 2019, the second highest over the entire 24-year time series. **(c)** Time series of the number of anticyclonic (red line) and cyclonic (blue line) eddies detected during December between 1997 – 2020. The broad propagation of the bloom is consistent with regional mesoscale eddy bloom dispersion in the Madagascar basin. Monthly climatological averages of Eddy Kinetic Energy (EKE), computed within the bloom area, reach maximum values between October – December, coinciding with the onset of the 2019/2020 bloom. EKE in December 2019 was the second highest observed over the last 23 years (1997 – 2020) and was predominantly associated with anticyclonic eddy activity. Accordingly, strong EKE (indicative of high eddy diffusivity) in December 2019 contributed to the diffusion of biomass westwards into the Mozambique channel and eastwards towards the Madagascar basin (Fig. 1). Additionally, the prevalence of anticyclonic eddies over cyclonic eddies means that the eddy field primarily had a dispersive role, as opposed to stimulating phytoplankton growth via the upward flux of nutrients from deeper layers(*11*). **(d)** Spatial map of the average eddy kinetic energy (EKE) for November 2019 – January 2020 over the Madagascar bloom area and broader southwest Indian Ocean. Strong kinetic energy during the bloom period contributed to the diffusion of fertilized waters into the Mozambique channel and Madagascar basin.

^\^


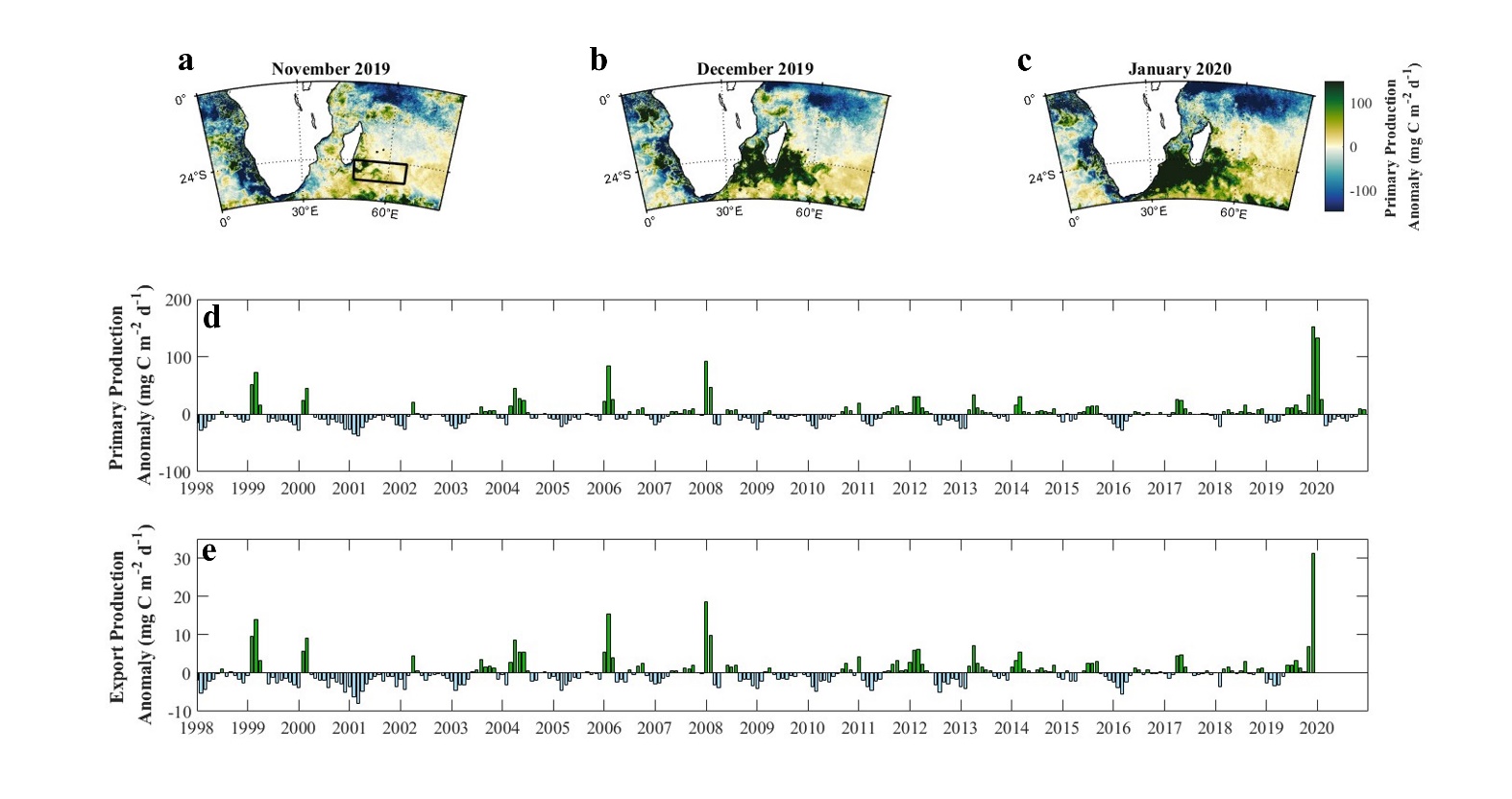


**Fig. S2** **(a-c)** Monthly anomalies of primary production over the broader Southern Indian Ocean. The black rectangle highlights the bloom area (24-30 ^o^S; 48-66 ^o^E) used for the computation of spatial averages (**d)** Monthly anomalies of primary production spatially averaged over the bloom area (see black rectangle in left panel of (a), for the period between 1998 - 2021. (**e)** Monthly anomalies of export production spatially averaged over the bloom area (see black rectangle in left panel of (**a**), for the period between 1998 - 2020. Future alterations to primary productivity may disrupt the Ocean Biological Carbon Pump (OBCP), a key mechanism of carbon sequestration that modulates the exchange of carbon dioxide (CO_2_) between the ocean and atmosphere(*12*), and ultimately, atmospheric CO_2_ concentrations. Although previously characterized as a region where air-sea CO_2_ fluxes are at near-equilibrium, in 2019/2020 the Madagascar bloom was a strong CO_2_ sink.


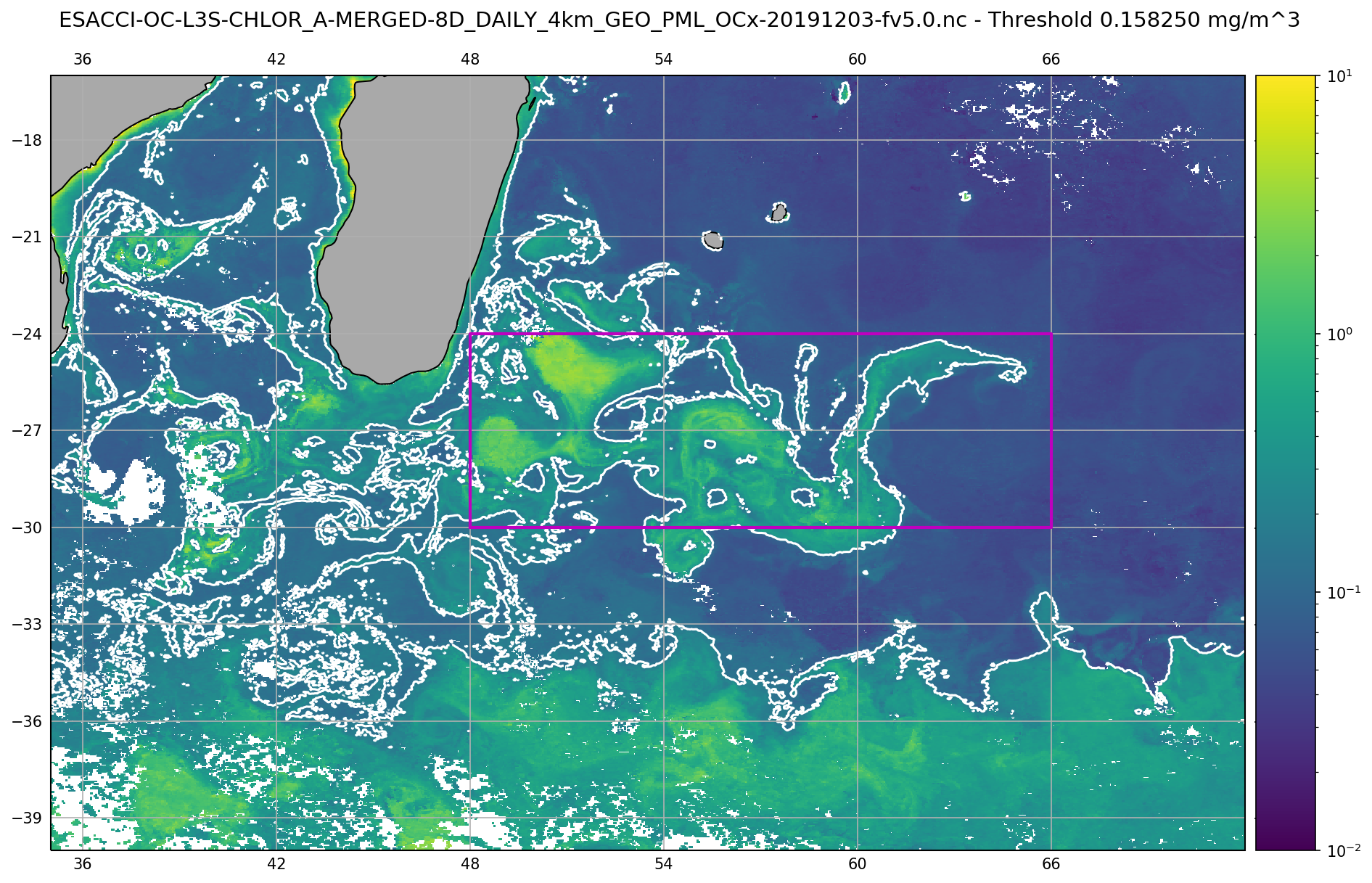


**Fig. S3.** OC-CCI 8-day composite surface Chl-a concentration for 3^rd^ December 2019. The magenta box identified the area of focus where particles were deployed to reconstruct the 90-day backward Lagrangian Trajectories. The white contours mark the boundaries of the bloom identified via the threshold of 0.158 mg m^-3.


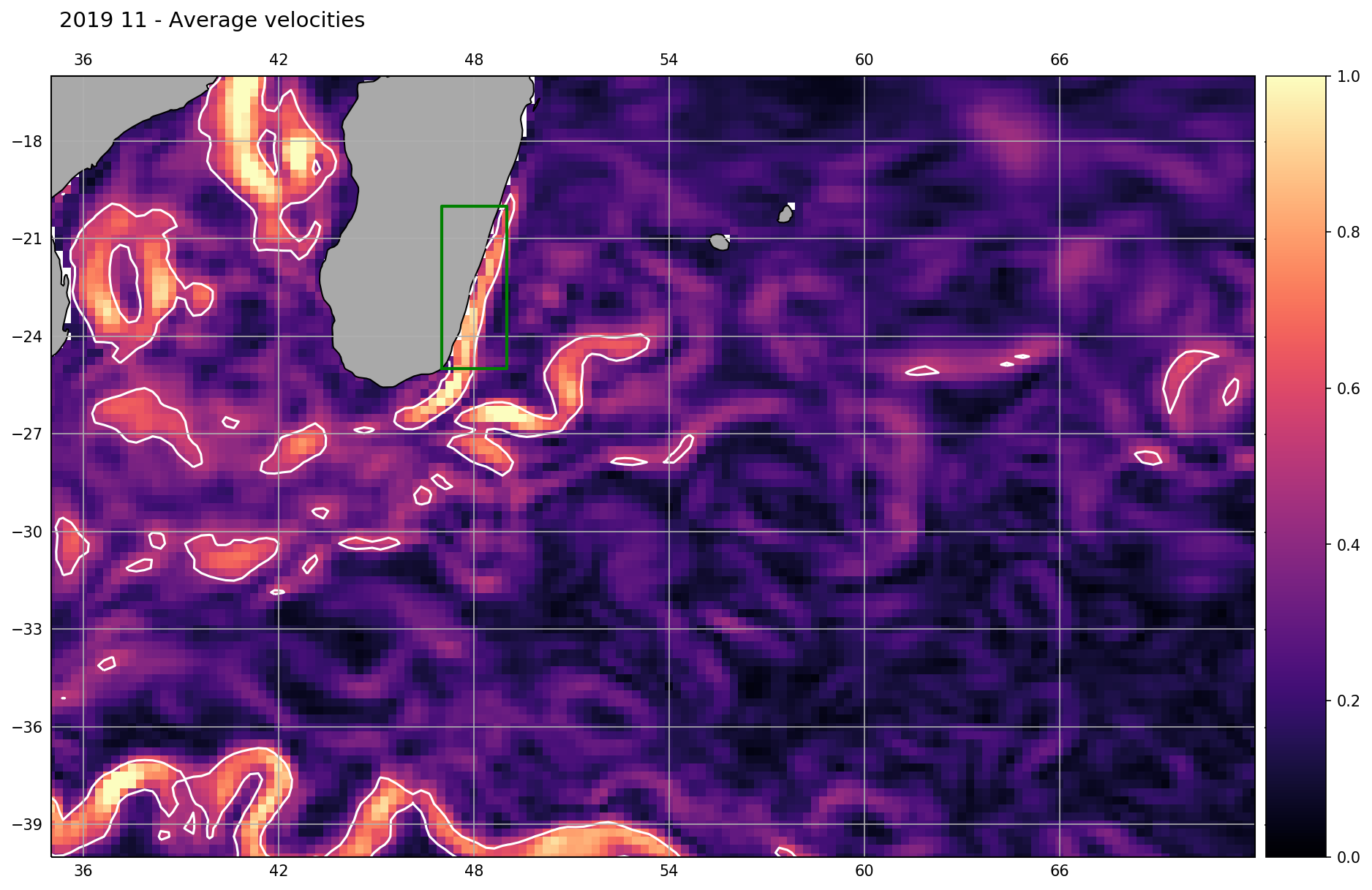


**Fig. S4.** Average surface geostrophic velocities for November 2019. The white contours mark the 0.5 m/s threshold. Particles coming from the region of high velocities within the green box (47 to 49 East and 20 to 25 South) are assumed to carry nutrient-rich waters from the Eastern Madagascar shelf.


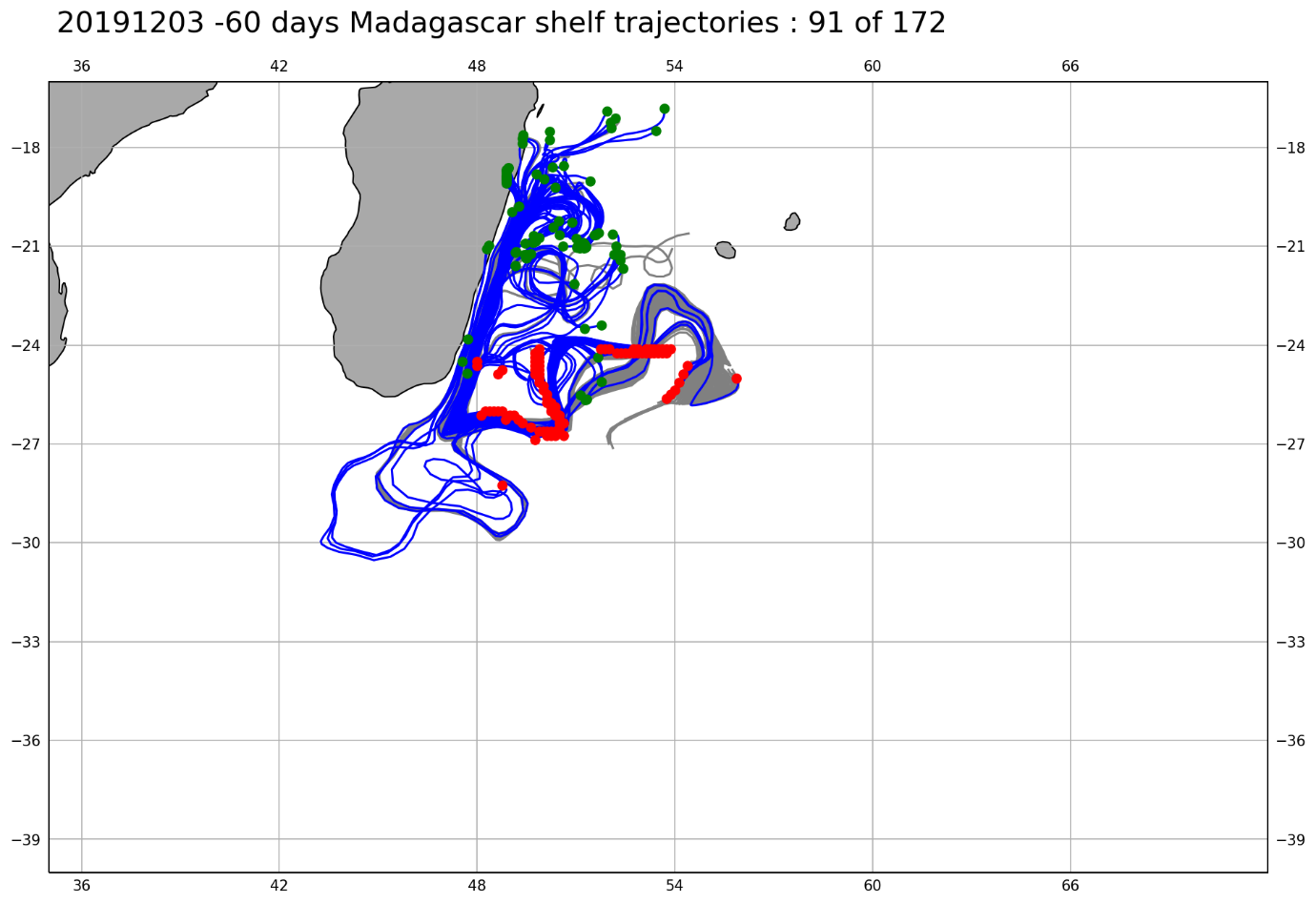


**
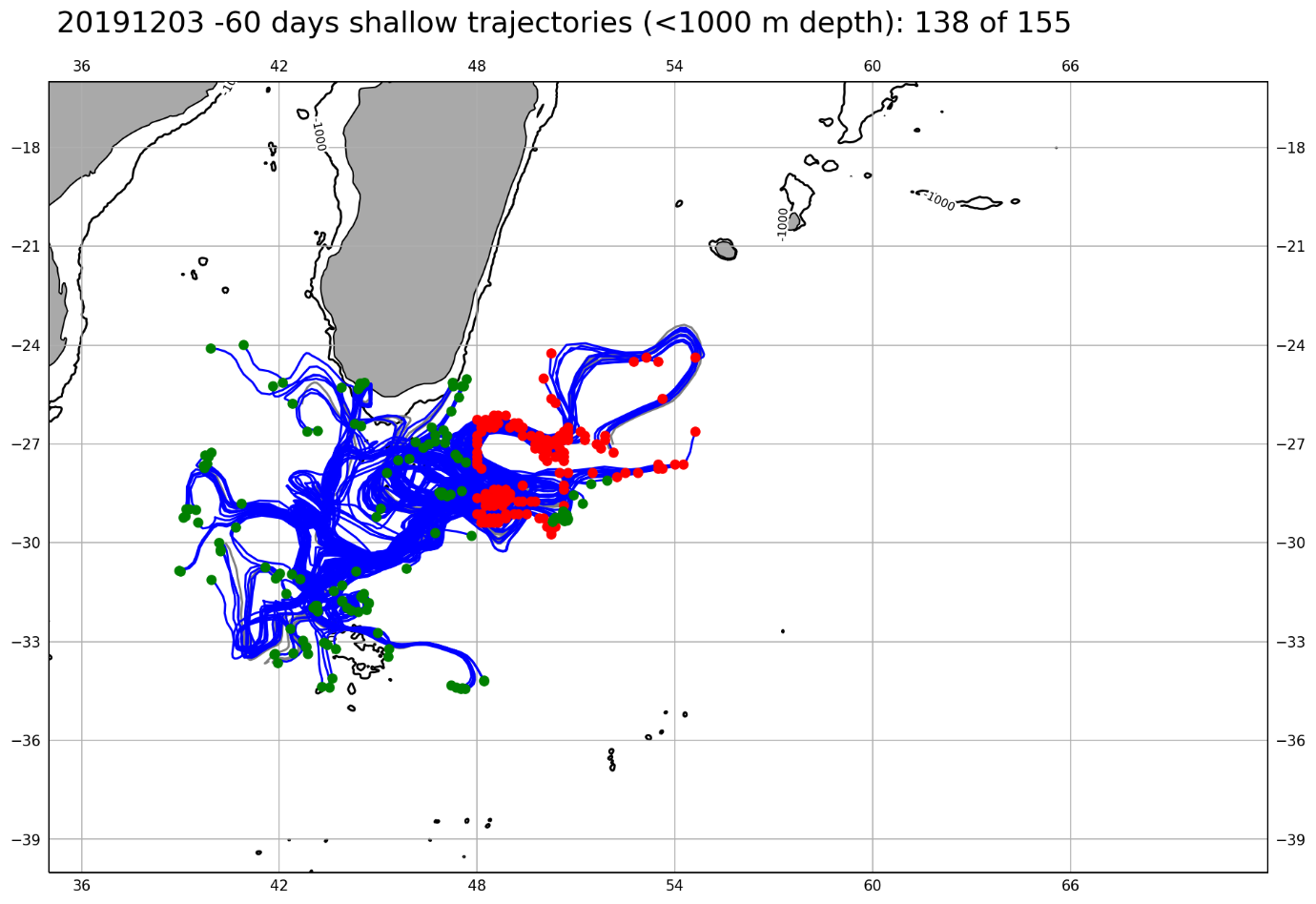
**

**
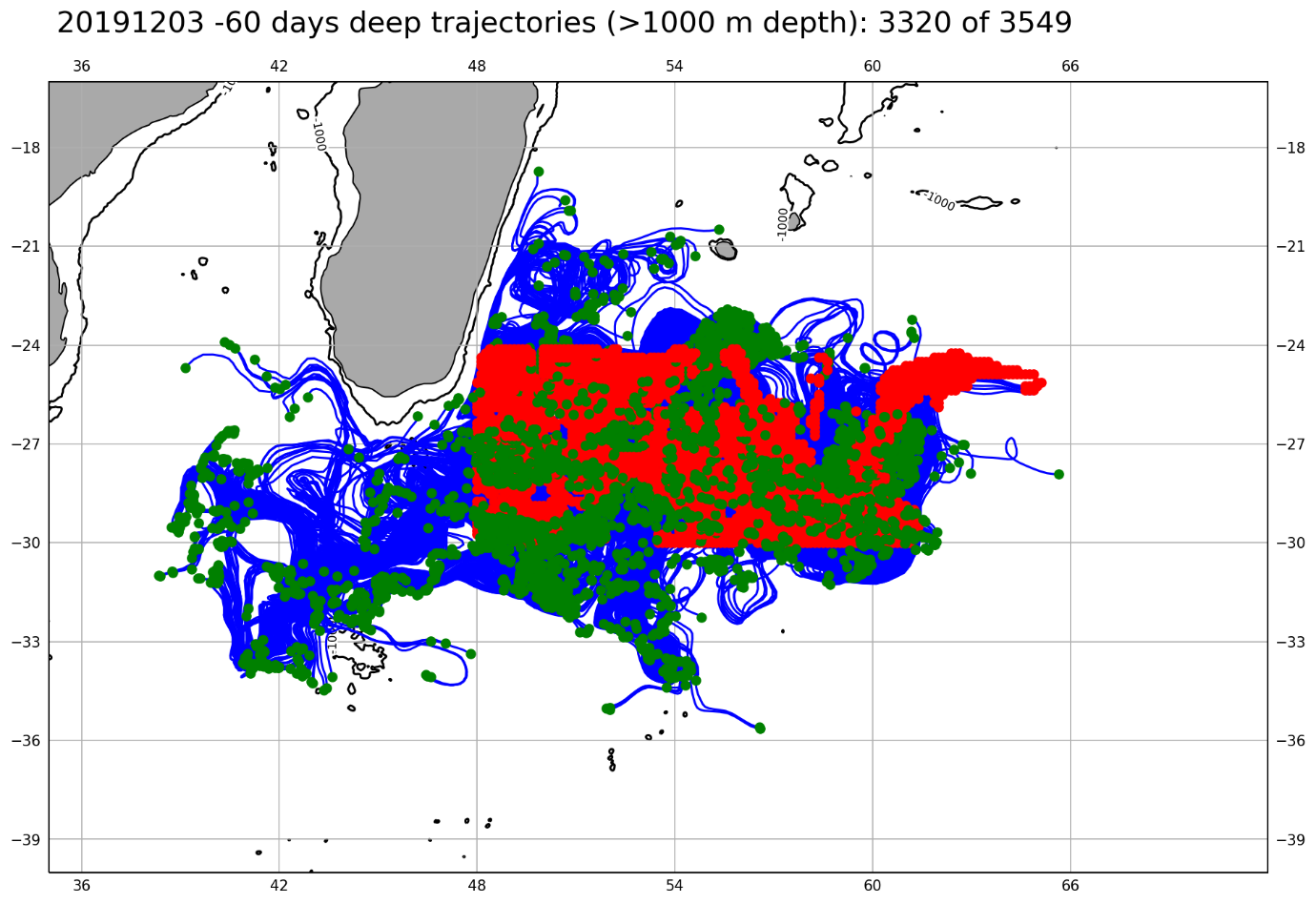
**

**Fig. S5**. 60-day backward Lagrangian trajectories for particles initially deployed within the region of focus (48° to 66° East and 24° to 30° South) on December 3. (Top) Trajectories from the East Madagascar Shelf; (middle) Trajectories from south-eastern shelves; (bottom) trajectories from the open ocean. Red dots indicate the position of particle release on December 3; green dots indicate their origin position 60 days before. The trajectories of particles within the bloom on December 3 are in blue; the trajectories of particles outside the bloom are in gray. For figure clarity, only the trajectories within the bloom are shown in the bottom panel.


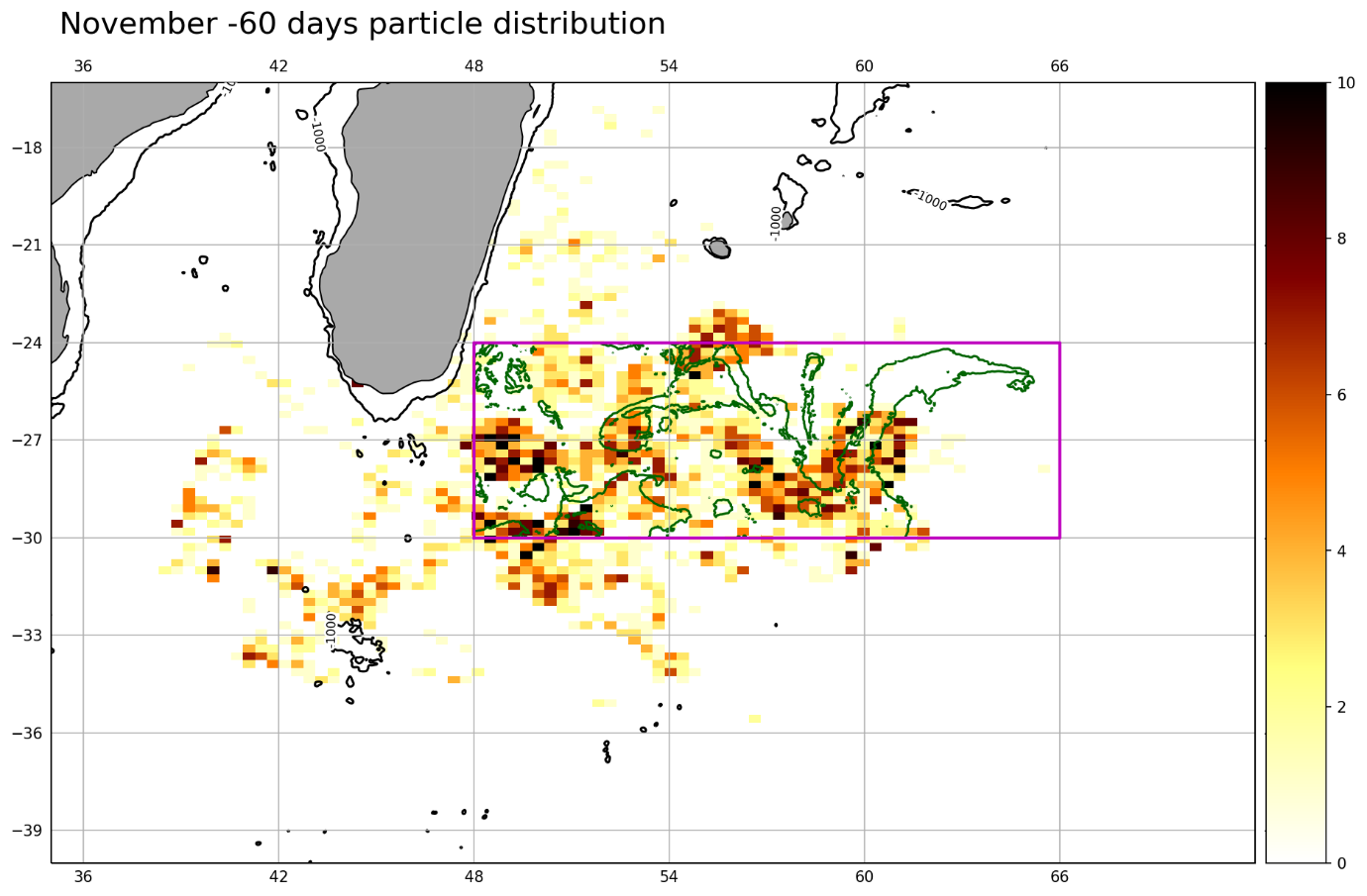


**Fig. S6.** Distribution of the particles within the bloom 60 days before December 3^rd^ 2019. Particles have been binned into a 100x100 bin grid spanning the figure domain. The magenta box indicates the area of focus of the study. The green contour marks the 0.158 mg m^-3^ Chl-a concentration threshold delimiting the bloom within which the particles were initially deployed before the backward advection.


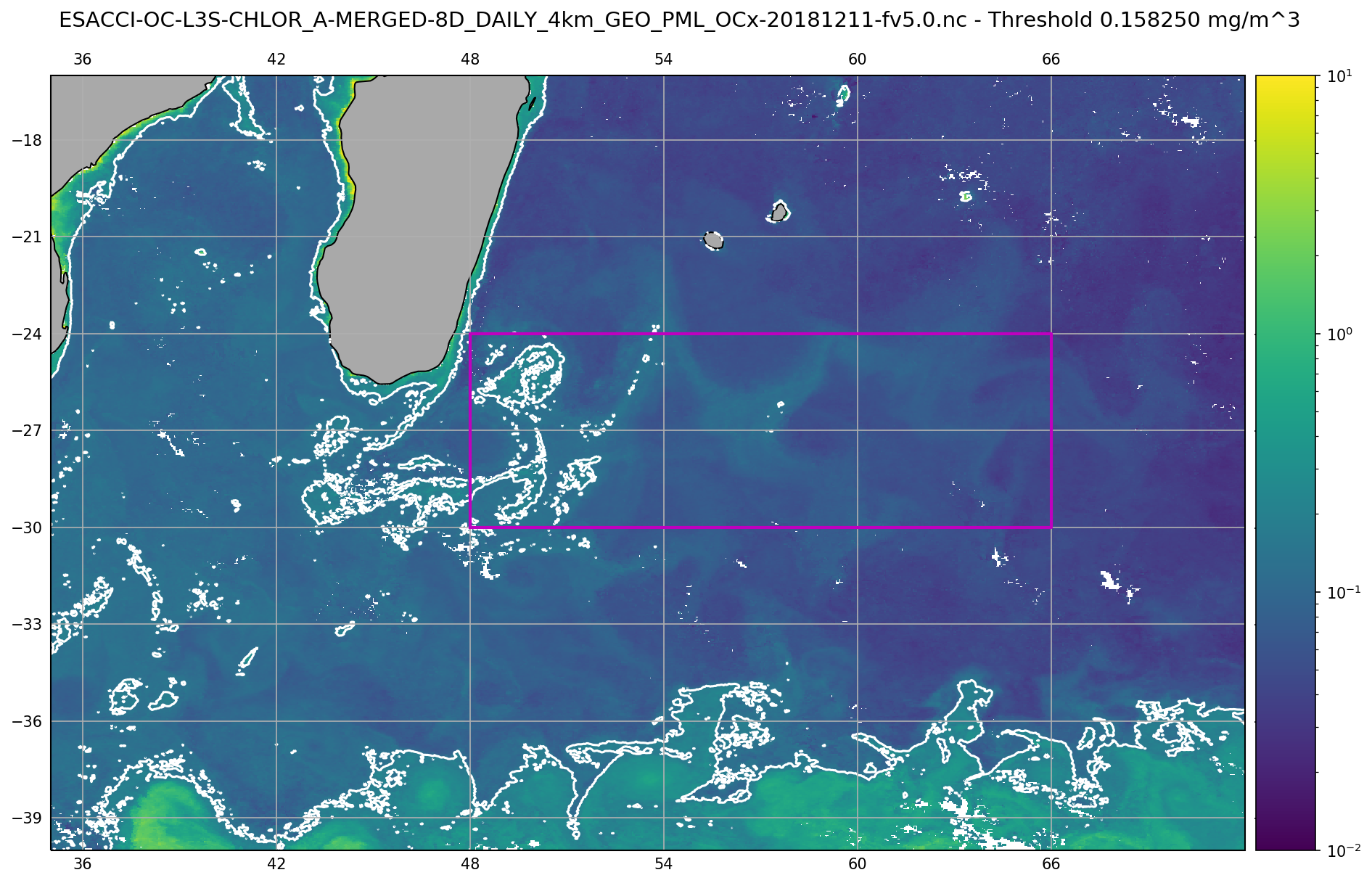


**Fig. S7.** Same as Supplementary Figure 3 but for 11^th^ December 2018.


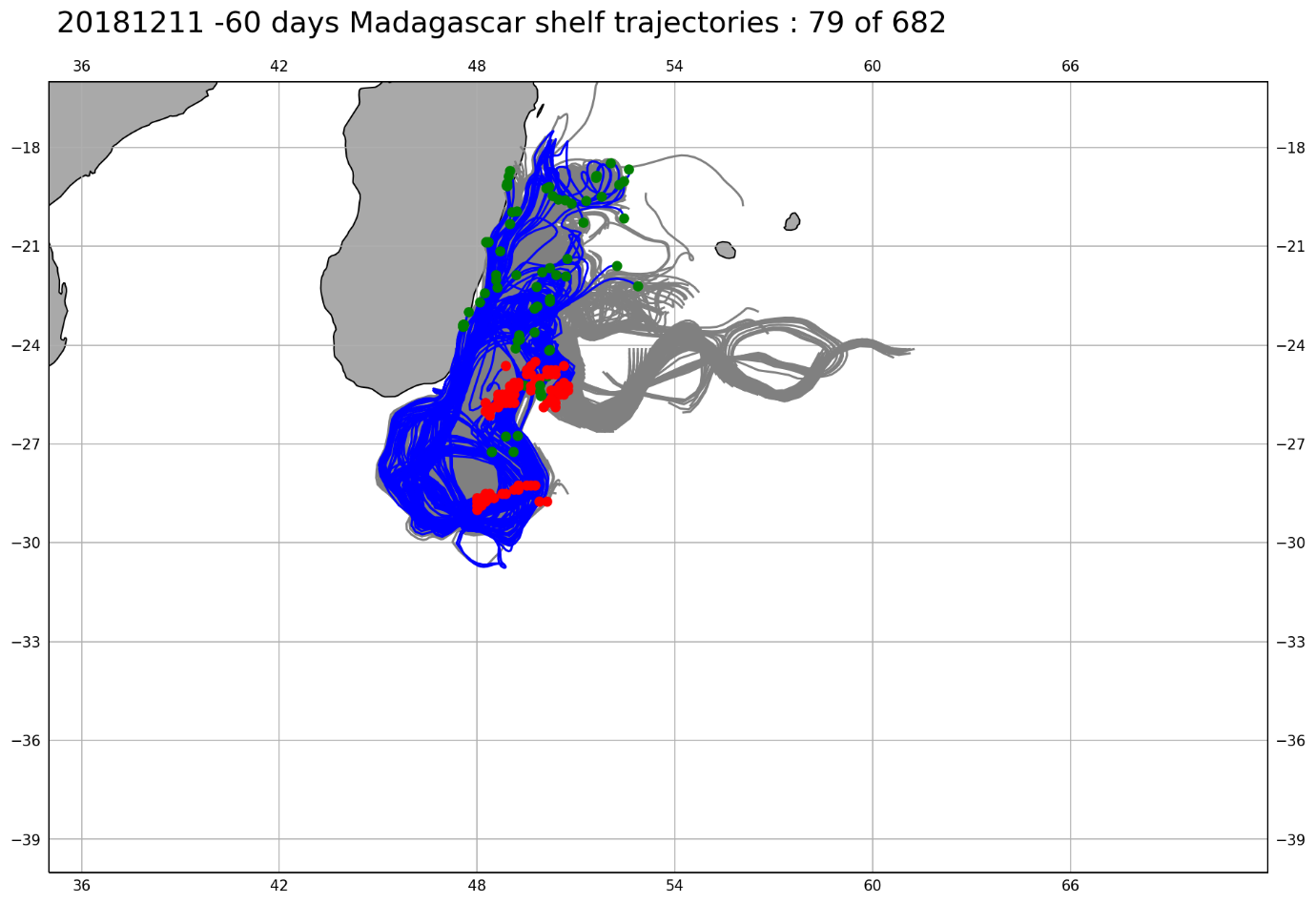


**
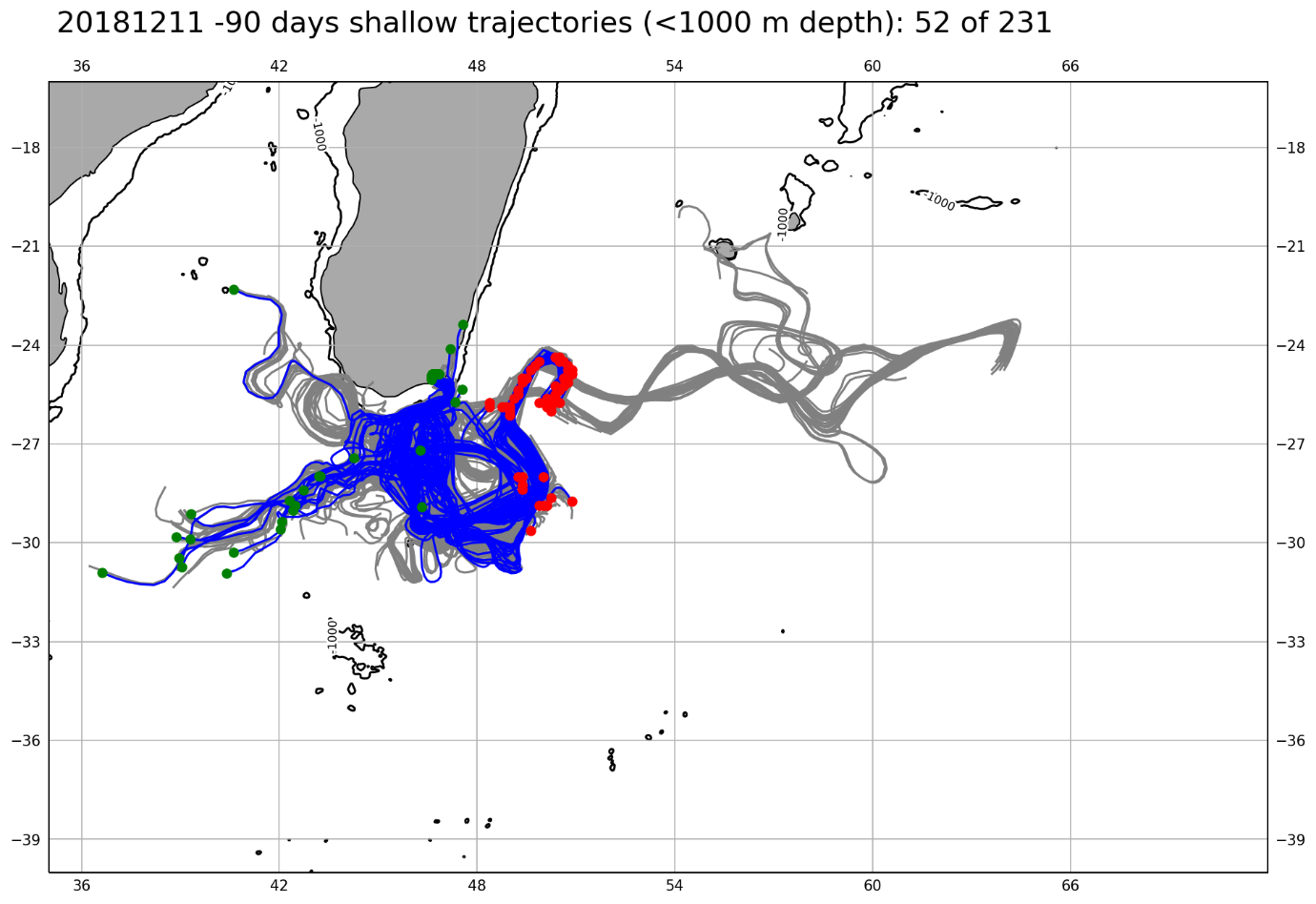
**

**Fig. S8.** Same as Supplementary Figure 5, top and middle, but for 11^th^ December 2018.


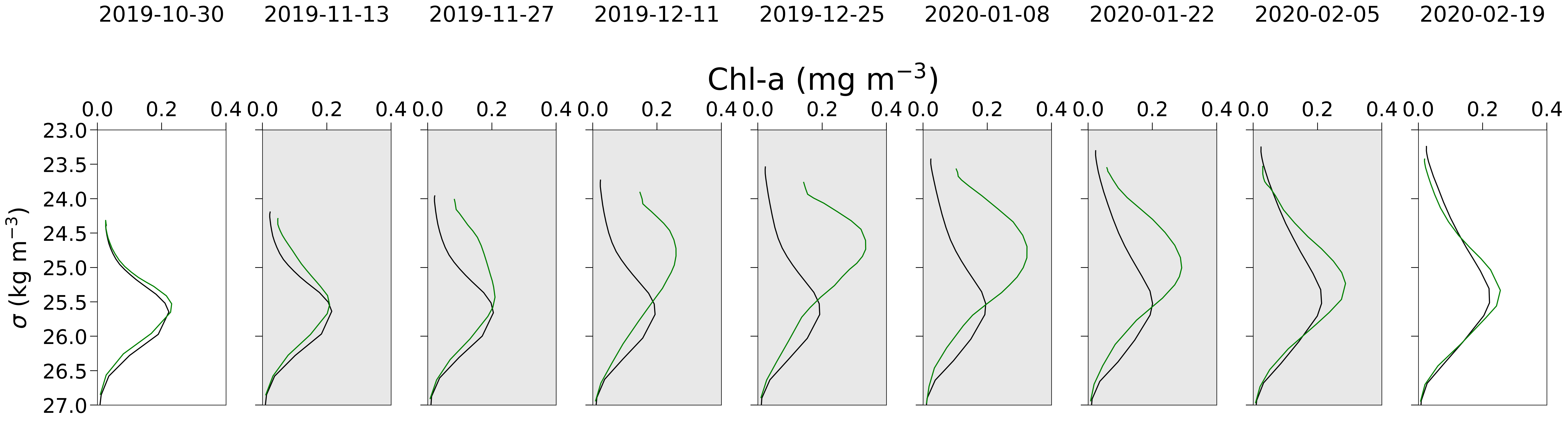

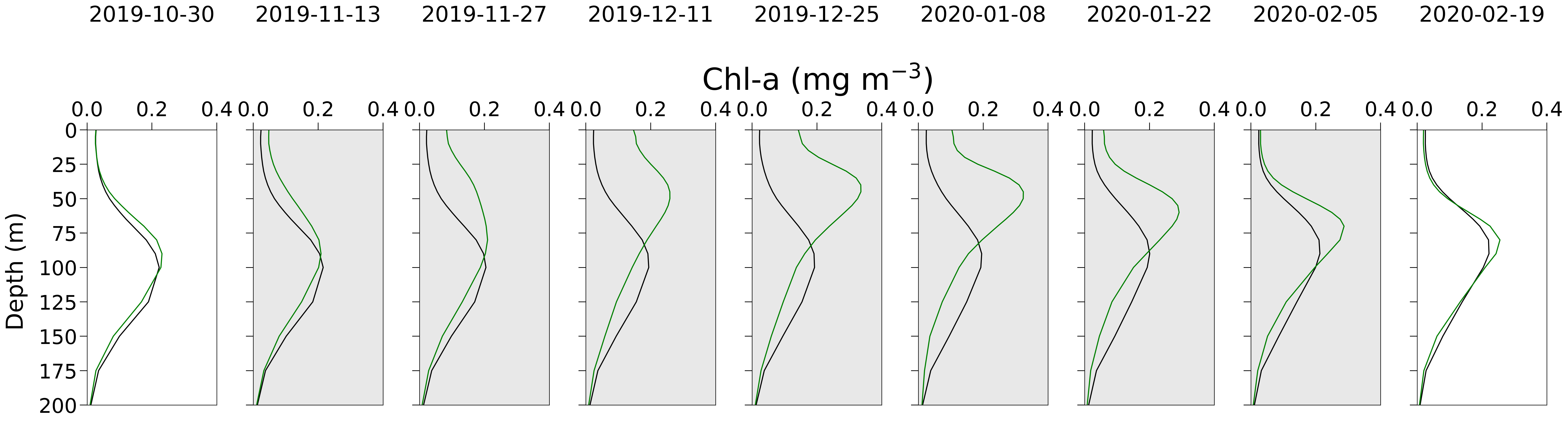

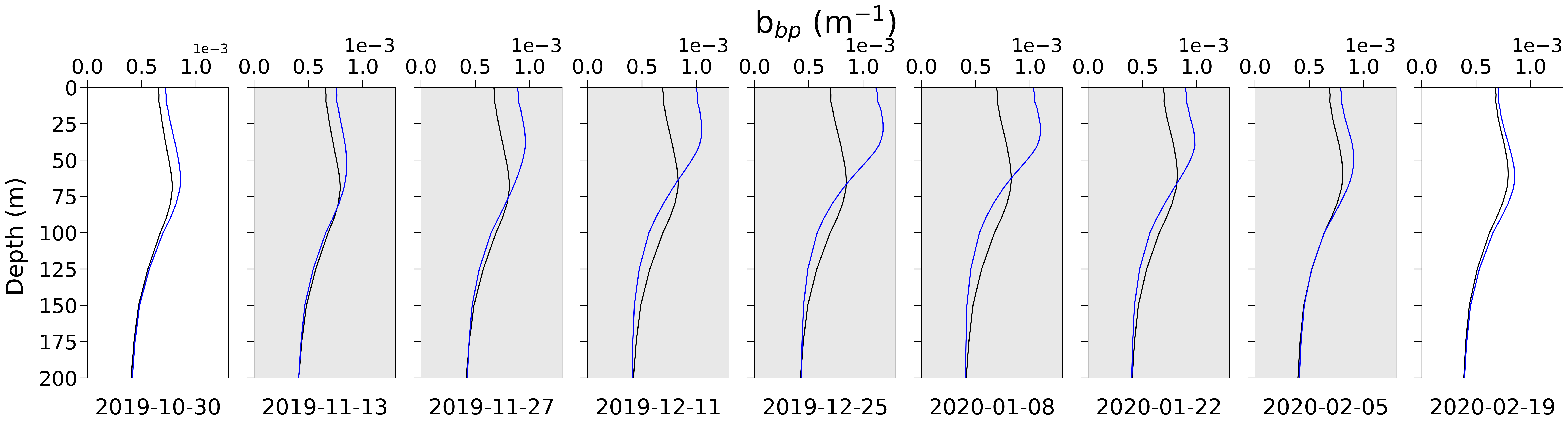

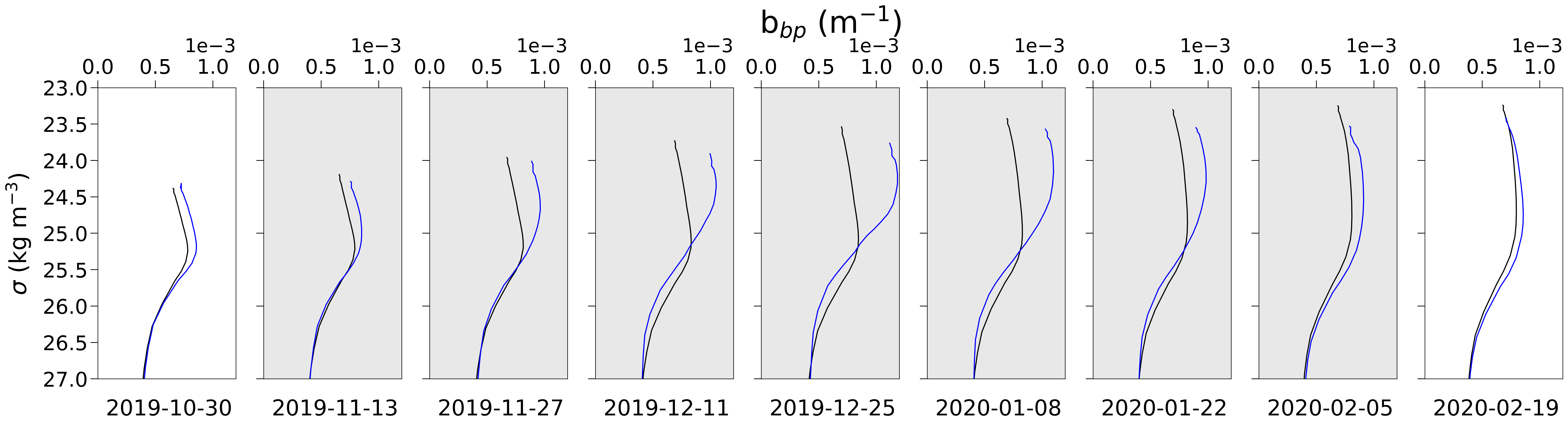


(a)

(b)

(c)

(d)

**Fig. S9.** Depth profiles of **(a)** Chl-a (green lines) and **(b)** particulate backscattering coefficient (b_bp,_ blue lines) throughout the 2019-2020 bloom period. The respective climatological profiles of each parameter are shown with black lines. **(c)** as shown in **(a)** with Chl-a concentration plotted versus density. **(d)** as shown in **(b)** with b_bp_ plotted versus density. Data have been averaged over the bloom box and are presented as bi-weekly mean values. Highlighted panels correspond to the bloom duration.


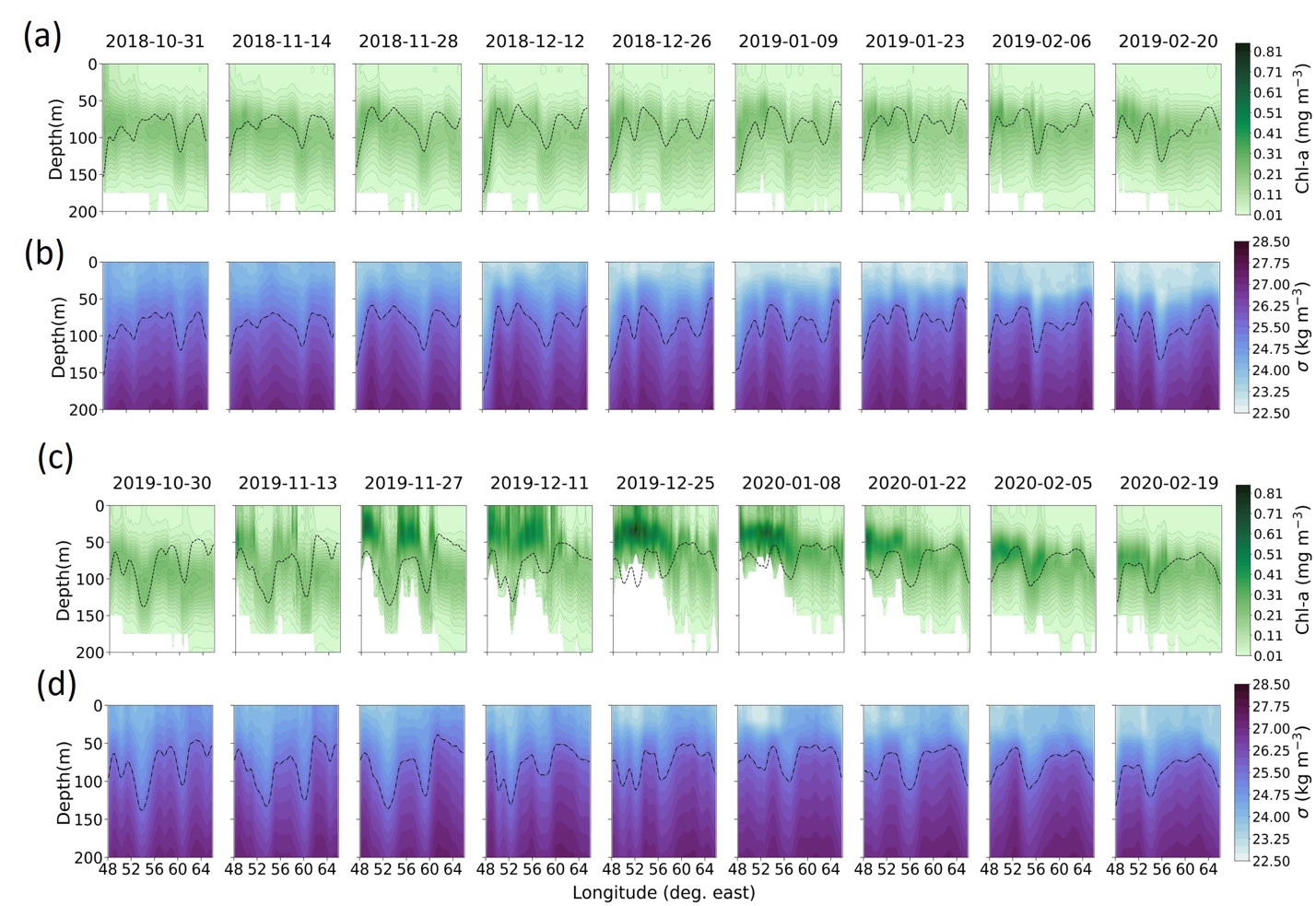


**Fig. S10.** Vertical longitudinal transects (48-66 ^o^E, 27-27.5 ^o^S) of Chl-a **(a, c)** (Chl-a) and density (σ) **(b, d)** during November 2018 - February 2019 non-bloom period (a, b) and November 2019 - February 2020 bloom event (c, d). Dates on top of Chl-a panels correspond to the starting date of temporal mean. The dashed line marks the 25.5 kg m-3 isopycnal.


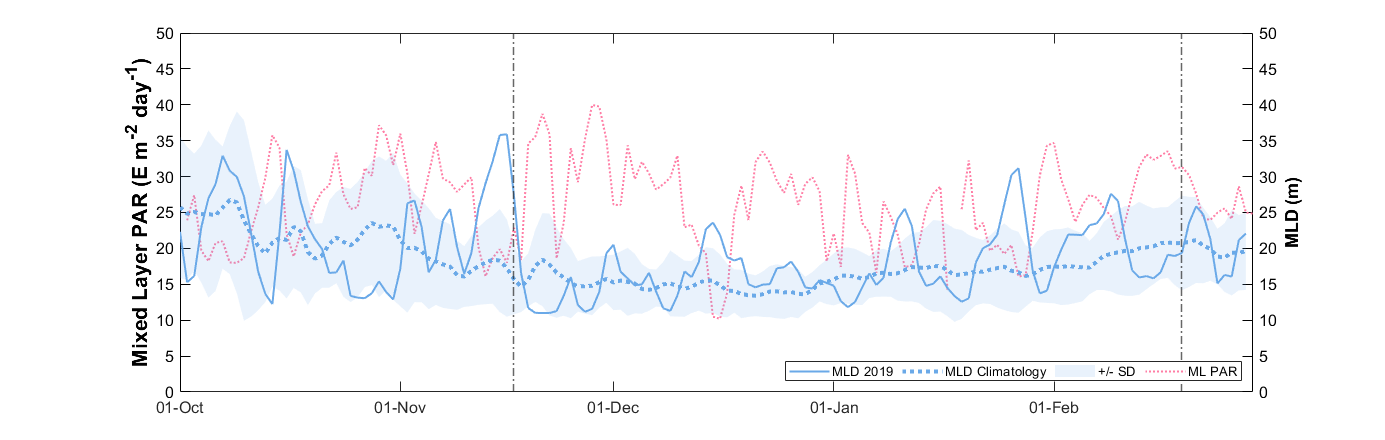


**Fig. S11.** Daily time series of Mixed Layer Depth (MLD) (Mercator GLORYS Ocean Reanalysis) with Photosynthetically Active Radiation (MODIS PAR) computed within the mixed layer (ML-PAR, pink dashed line). Time series are based on the area-averaged variables over the defined Madagascar bloom area (see Fig. 1a). The solid blue line represents the daily MLD time series for austral spring/summer of 2019/2020, whilst the shaded areas represent +/- standard deviation. The daily MLD climatology is given by the blue dashed line. Overall, ML-PAR remained generally consistent throughout both prior and post bloom initiation, indicating that light availability was not a limiting factor on bloom development.

**
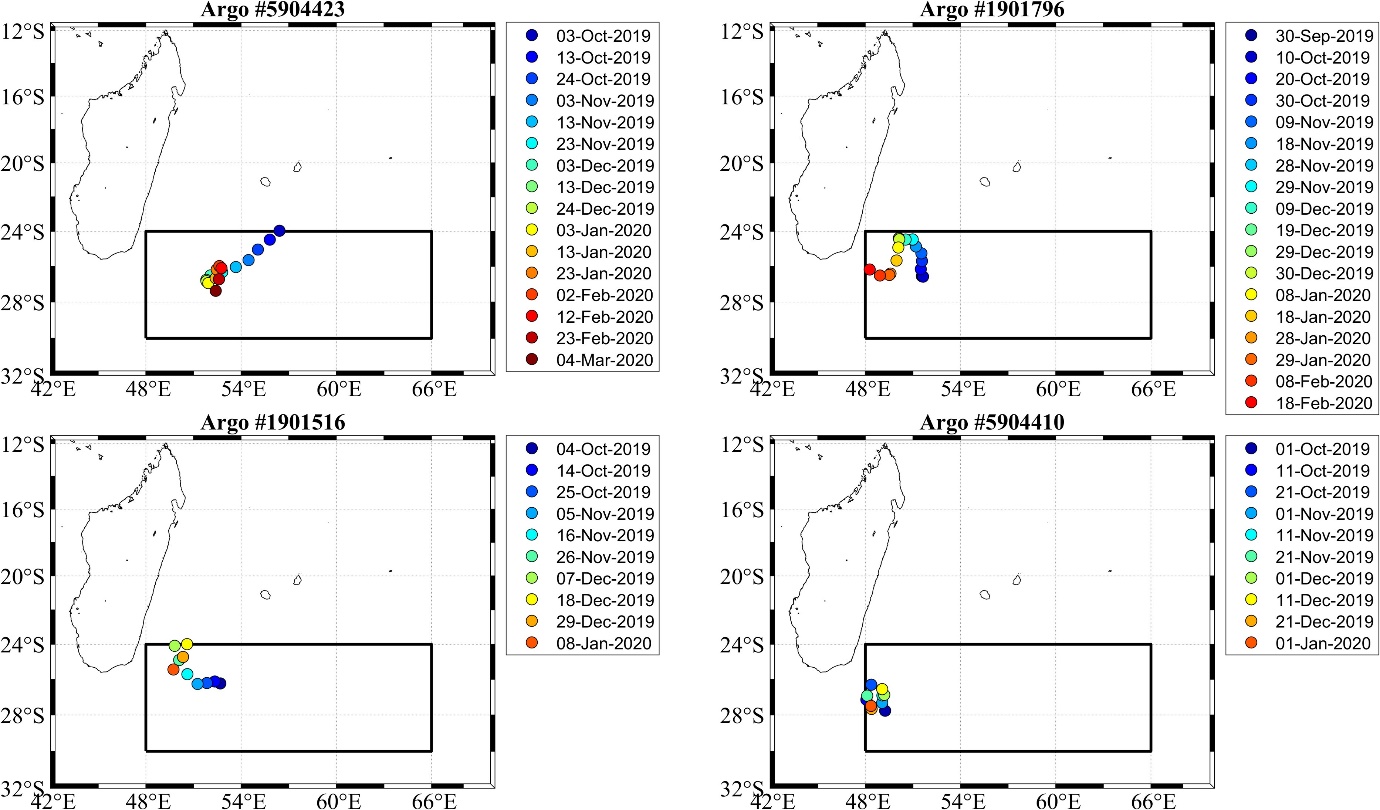
**

**Fig. S12.** Maps showing the locations of the four core Argo floats within the northwest part of the bloom area. *In situ* mixed layer temperature data computed using these Argo profiles revealed colder temperatures prior to the initiation of the austral spring/summer 2019/2020 phytoplankton bloom, followed by a rapid warming which continued throughout December 2019 and January 2020 (Fig. 2d). This analysis is concurrent with time series and spatial composites of satellite-derived SST (Fig. 2d, Supplementary Fig. 13).

**
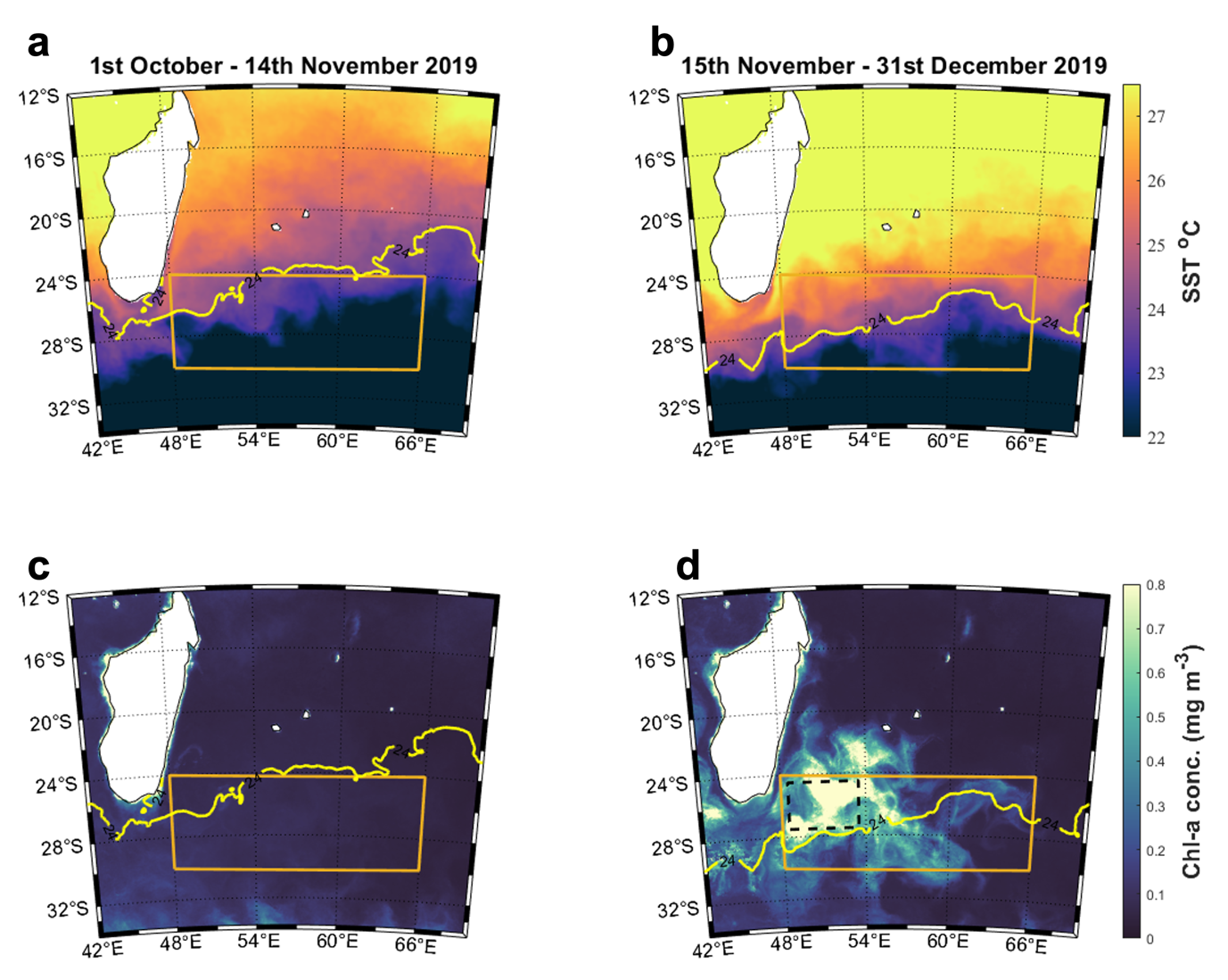
**

**Fig. S13.** **(a)** Spatial composites of sea surface temperature (OSTIA-SST) averaged between 1^st^ October 2019 - 14^th^ November 2019. This period was selected to represent regional surface temperature conditions before the initiation of the 2019/2020 Madagascar austral spring/summer phytoplankton bloom. **(b)** As shown in (a) but encompassing the period 15^th^ November 2019 – 1^st^ January 2020. This period was selected to represent regional surface temperature conditions during the bloom initiation until its peak in late-December 2019. **(c-d)** Equivalent temporal composites of Chl-a concentration. The yellow contour line in each plot represents the 24^o^C isotherm. The bloom area is depicted by the orange rectangle in each panel. The box highlighted by the dashed black line in panel **d** represents the area selected for the computation of the SST time series presented in Figure 2d (27.5 °S - 24.5°S, 48.5°E – 53.5°E). This region was selected to represent the region where the 2019/2020 bloom initiated. Prior to the bloom initiation, SST across almost the entire bloom area was characterized by colder temperatures (< 24 ^°^C) and low Chl-a concentrations. Between the bloom initiation on 15^th^ November 2019 and its peak at the end of December (panel **d**), a large northwest region within the bloom area subsequently experienced a rapid increase in SST, alongside maximum (> 0.8 mg m^-3^) Chl-a concentrations. This is spatially consistent with the position of the two mesoscale eddies that marked the start of the bloom in November 2019 (Fig. 1a).

**
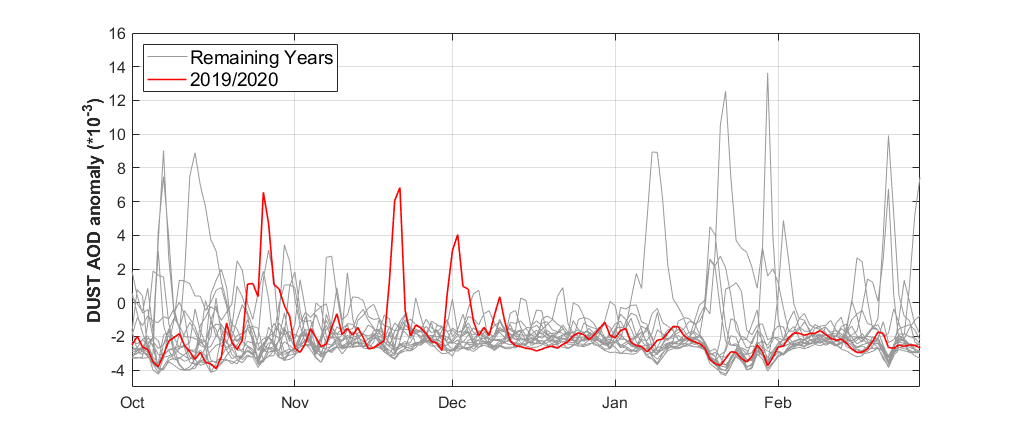
**

**Fig. S14.** Daily anomalies of Dust AOD (CAMS ECMWF) averaged over the bloom area between October - March for the year 2019 (red line). Corresponding daily time series of Dust AOD for the remaining years are shown by the grey lines. Dust AOD in mid-November (the approximate timing of the bloom initiation) and early December were unprecedently high, relative to the equivalent period for other years.

**
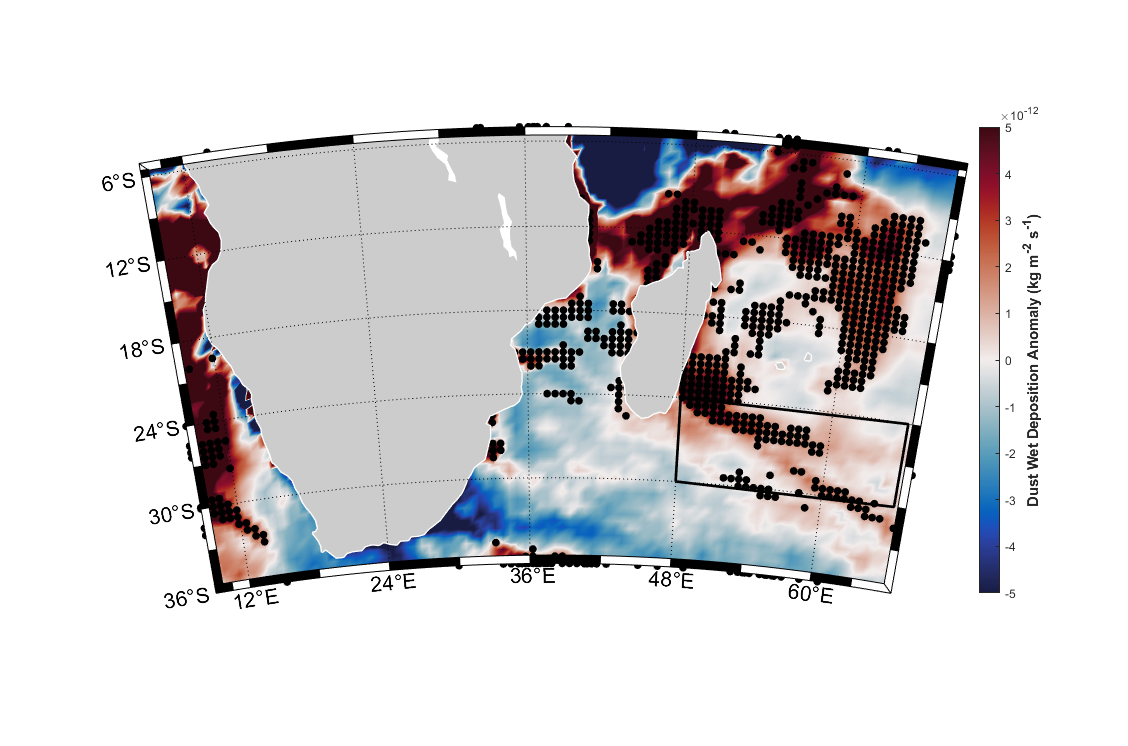
**

**Fig. S15.** Spatial composite of averaged daily anomalies of total dust aerosol wet deposition from November 15^th^ – December 31^st^, 2019 (NASA MERRA-2). The solid black circles represent locations where daily values of dust wet deposition were ≥ 4.5 standard deviations above climatological values on at least one day between November 15^th^ – December 31^st^, 2019. The black rectangle represents the defined Madagascar bloom area. Land masses have been masked to highlight dust aerosol wet deposition that occurred over the ocean.

**
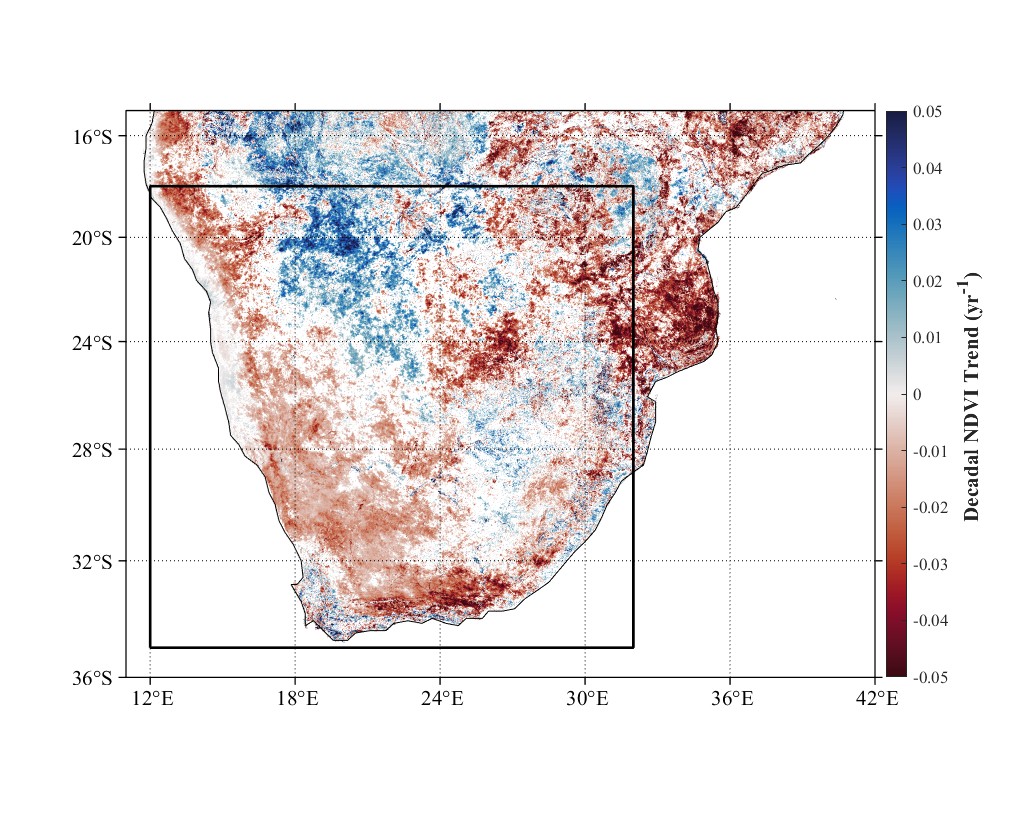
**

**Fig. S16.** Decadal trend in the Normalized Differenced Vegetation Index (NDVI, MODIS-Terra) over broader Southern Africa computed between February 2002 – December 2020. Values with a *p-value* > 0.05 have been masked. The black rectangle represents the area limits of broader Southern Africa utilized for the spatiotemporal analysis presented in Figure 4. A significant reduction in vegetation cover (as proxied by the NDVI) has occurred over large parts of western Southern Africa, encompassing parts of South Africa, Botswana, and Namibia – key dust sources areas identified in this study. Reduced vegetation cover is known to enhance the likelihood of wind-driven soil erosion and increase dust emissions in dryland areas.

**a**

**b**


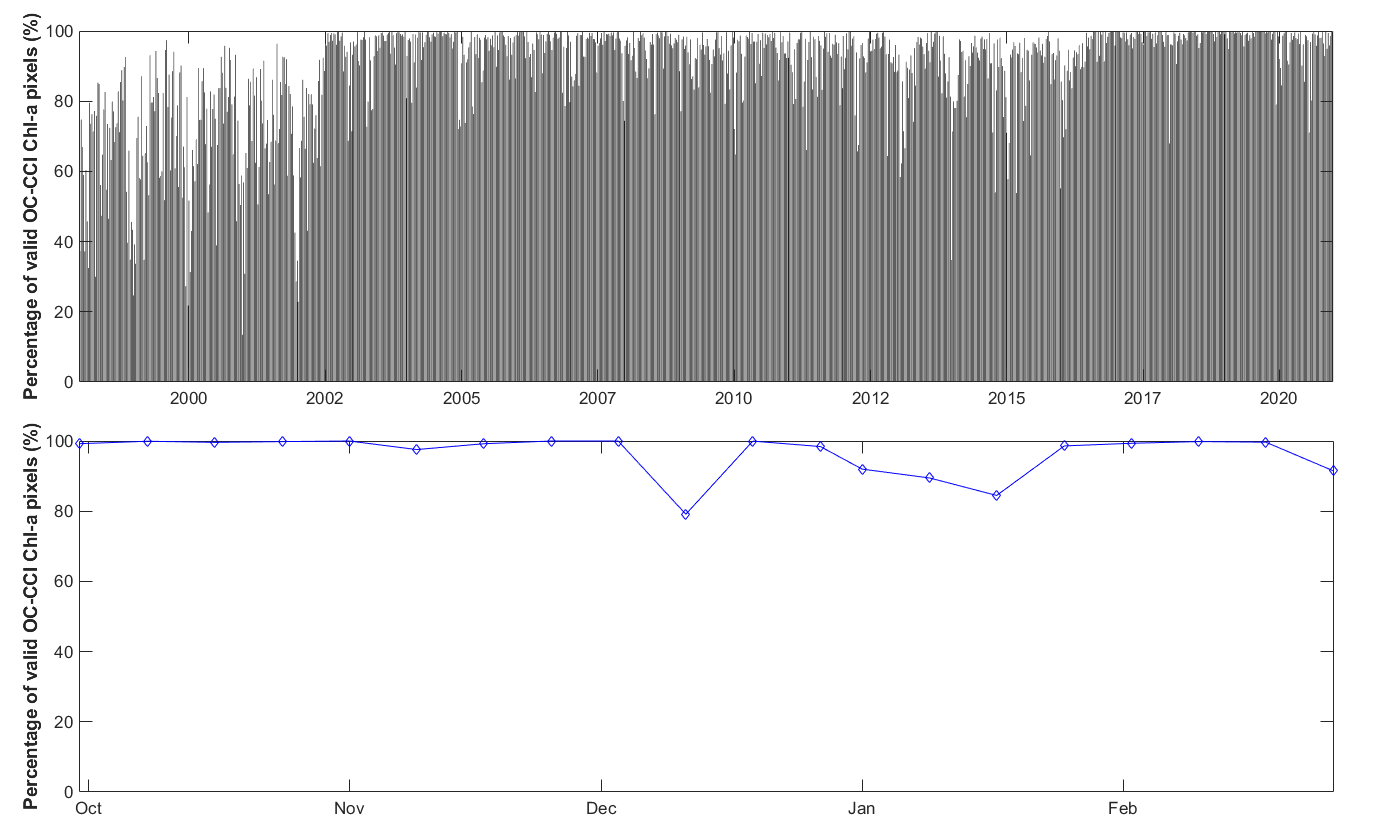


**Fig. S17. (a)** The percentage of valid Chl-a retrievals from the 8-day OC-CCI product over the Madagascar bloom area between 1998 – 2020 **(b)** The percentage of valid retrievals over the Madagascar bloom area during the austral spring/summer of 2019/2020.

**Fig. S18 (a**) Chlorophyll-a [Chl-a] time-series was calculated in 3,731 of 5° by 5° boxes from 1997 to 2020 in the broader Southwest Indian Ocean (15°S–40°S; 30°E–80°E). Yellow circles and yellow dashed boxes are examples to show the centre and coverage of each box region. Box moves by 1° eastward and southward sequentially illustrated by the black arrows. Box position 1, 91, 3,641 and 3,731 denoting the edge of the study region are shown as examples on the map of 2019 December Chl-a. The ratio of monthly Chl-a to its monthly climatology is calculated for each 5° by 5° box starting from September 1997 to December 2020. Black circles: centre locations of 5° by 5° boxes where $\frac{monthly [Chla]}{\left[ Chla \right]climatology}$ >3 before the 2019–2020 austral summer bloom (from September 1997 to August 2019); red circles: centre locations of 5° by 5° boxes where $\frac{monthly [Chla]}{\left[ Chla \right]climatology}$>3 during or after the 2019–2020 austral summer bloom. Large region of the Southwest Indian Ocean showed unprecedented Chl-a concentration during 2019-2020 austral summer. **b**, Ratio of monthly Chl-a to its corresponding monthly climatologies for each box region from 1997 to 2020. **c**, Frequency distributions of the monthly Chl-a to monthly climatology ratios over the historical and 2019–2020 austral summers.

**Supplementary Movie 1**. Daily progression of dust AOD from Southern Africa towards the southeast Madagascar Sea during the austral spring/summer of 2019.

**References for Supplementary Material**

1. E. van Sebille, S. M. Griffies, R. Abernathey, T. P. Adams, P. Berloff, A. Biastoch, B. Blanke, E. P. Chassignet, Y. Cheng, C. J. Cotter, E. Deleersnijder, K. Döös, H. F. Drake, S. Drijfhout, S. F. Gary, A. W. Heemink, J. Kjellsson, I. M. Koszalka, M. Lange, C. Lique, G. A. MacGilchrist, R. Marsh, C. G. Mayorga Adame, R. McAdam, F. Nencioli, C. B. Paris, M. D. Piggott, J. A. Polton, S. Rühs, S. H. A. M. Shah, M. D. Thomas, J. Wang, P. J. Wolfram, L. Zanna, J. D. Zika, Lagrangian ocean analysis: Fundamentals and practices. *Ocean Model.* **121**, 49–75 (2018).
2. F. Nencioli, F. D’Ovidio, A. M. Doglioli, A. A. Petrenko, Surface coastal circulation patterns by in-situ detection of Lagrangian coherent structures. *Geophys. Res. Lett.* **38** (2011).
3. F. D’Ovidio, A. Della Penna, T. W. Trull, F. Nencioli, M. I. Pujol, M. H. Rio, Y. H. Park, C. Cotté, M. Zhou, S. Blain, The biogeochemical structuring role of horizontal stirring: Lagrangian perspectives on iron delivery downstream of the Kerguelen Plateau. *Biogeosciences* **12**, 5567–5581 (2015).
4. F. Nencioli, G. Dall’Olmo, G. D. Quartly, Agulhas Ring Transport Efficiency From Combined Satellite Altimetry and Argo Profiles. *J. Geophys. Res. Oceans* **123**, 5874–5888 (2018).
5. J. J. Nauw, H. M. van Aken, A. Webb, J. R. E. Lutjeharms, W. P. M. de Ruijter, Observations of the southern East Madagascar Current and undercurrent and countercurrent system. *J. Geophys. Res. Oceans* **113**, 8006 (2008).
6. J. D. Ramanantsoa, P. Penven, R. P. Raj, L. Renault, L. Ponsoni, M. Ostrowski, A. F. Dilmahamod, M. Rouault, Where and How the East Madagascar Current Retroflection Originates? *J. Geophys. Res. Oceans* **126**, e2020JC016203 (2021).
7. N. P. Fofonoff, R. C. Jr. Millard. Algorithms for Computation of Fundamental Properties of Seawater. Endorsed by UNESCO/SCOR/ICES/IAPSO Joint Panel on Oceanographic Tables and Standards and SCOR Working Group 51. *Unesco Technical Papers in Marine Science*, **44** (1983).
8. F. J. Millero, C. T. Chen, A. Bradshaw, K. Schleicher, A new high pressure equation of state for seawater. *Deep Sea Res. Part I: Oceanogr. Res. Pap.* **27**, 255–264 (1980).
9. P. M. Saunders, Practical Conversion of Pressure to Depth. *J. Phys. Oceanogr.* **11**, 573–574 (1981).
10. M. A. Srokosz, G. D. Quartly, The Madagascar Bloom: A serendipitous study. *J. Geophys. Res. Oceans* **118**, 14–25 (2013).
11. D. J. McGillicuddy, A. R. Robinson, D. A. Siegel, H. W. Jannasch, R. Johnson, T. D. Dickey, J. McNeil, A. F. Michaels, A. H. Knap, Influence of mesoscale eddies on new production in the Sargasso Sea. *Nature* **394**, 263–266 (1998).
12. R. J. W. Brewin, S. Sathyendranath, T. Platt, H. Bouman, S. Ciavatta, G. Dall’Olmo, J. Dingle, S. Groom, B. Jönsson, T. S. Kostadinov, G. Kulk, M. Laine, V. Martínez-Vicente, S. Psarra, D. E. Raitsos, K. Richardson, M. H. Rio, C. S. Rousseaux, J. Salisbury, J. D. Shutler, P. Walker, Sensing the ocean biological carbon pump from space: A review of capabilities, concepts, research gaps and future developments. *Earth Sci. Rev.* **217**, 103604 (2021).
